# Measuring physical activity in older adults: calibrating cut-points for the MotionWatch 8^©^

**DOI:** 10.3389/fnagi.2015.00165

**Published:** 2015-08-25

**Authors:** Glenn J. Landry, Ryan S. Falck, Michael W. Beets, Teresa Liu-Ambrose

**Affiliations:** ^1^Aging, Mobility, and Cognitive Neuroscience Lab, Department of Physical Therapy, Faculty of Medicine, University of British ColumbiaVancouver, BC, Canada; ^2^Djavad Mowafaghian Centre for Brain Health, University of British ColumbiaVancouver, BC, Canada; ^3^Department of Exercise Science, Arnold School of Public Health, University of South CarolinaColumbia, SC, USA; ^4^Brain Research Centre, University of British ColumbiaVancouver, BC, Canada

**Keywords:** accelerometers, actigraphy, indirect calorimeters, validation, physical activity, older adults

## Abstract

Given the world’s aging population, the staggering economic impact of dementia, the lack of effective treatments, and the fact a cure for dementia is likely many years away – there is an urgent need to develop interventions to prevent or at least delay dementia’s progression. Thus, lifestyle approaches to promote healthy aging are an important line of scientific inquiry. Good sleep quality and physical activity (PA) are pillars of healthy aging, and as such, are an increasing focus for intervention studies aimed at promoting health and cognitive function in older adults. However, PA and sleep quality are difficult constructs to evaluate empirically. Wrist-worn actigraphy (WWA) is currently accepted as a valid objective measure of sleep quality. The MotionWatch 8^©^ (MW8) is the latest WWA, replacing the discontinued Actiwatch 4 and Actiwatch 7. In the current study, concurrent measurement of WWA and indirect calorimetry was performed during 10 different activities of daily living for 23 healthy older adults (aged 57–80 years) to determine cut-points for sedentary and moderate-vigorous PA – using receiver operating characteristic curves – with the cut-point for light activity being the boundaries between sedentary and moderate to vigorous PA. In addition, simultaneous multi-unit reliability was determined for the MW8 using inter-class correlations. The current study is the first to validate MW8 activity count cut-points – for sedentary, light, and moderate to vigorous PA – specifically for use with healthy older adults. These cut-points provide important context for better interpretation of MW8 activity counts, and a greater understanding of what these counts mean in terms of PA. Hence, our results validate another level of analysis for researchers using the MW8 in studies aiming to examine PA and sleep quality concurrently in older adults.

## Introduction

Worldwide, one new case of dementia is detected every 4 s (WHO and ADI, 2012). Given the world’s aging population, the staggering economic impact of dementia (Wimo and Prince, 2010), the lack of effective treatments, and the fact a cure for dementia is likely many years away – there is an urgent need to develop interventions to prevent or at least delay dementia’s progression (for review, see Landry and Liu-Ambrose, 2014). Thus, lifestyle approaches to promote healthy aging have become an important line of scientific inquiry.

Good quality sleep is a pillar of healthy aging. Unfortunately, sleep changes as a function of normal aging, both in terms of decreased quality and quantity (for reviews, see Espiritu, 2008; Crowley, 2011). Sleep complaints are common in older adults – more than half of adults 65+ have at least one chronic sleep complaint – the most common being an inability to stay asleep at night (Foley et al., 1995). Of critical importance is the growing evidence suggesting poor sleep quality is a contributing factor in the prevalence, progression, and severity of Alzheimer’s disease – the most common cause of dementia (Landry and Liu-Ambrose, 2014).

Physical activity (PA) is another important pillar of healthy aging. Epidemiological evidence suggests PA increases life span and reduces the risk of chronic conditions such as stroke, heart disease, dementia, and type-2 diabetes (Blair and Brodney, 1999; Nelson et al., 2007; Blair, 2009; Chodzko-Zajko et al., 2009; Hamer and Chida, 2009; Ahlskog et al., 2011; Erickson et al., 2012; Swift et al., 2013). Furthermore, PA in the form of exercise has long been thought to play an important role in the quality of sleep. Epidemiological studies examining the relationship between exercise and sleep (for reviews, see Driver and Taylor, 2000; Youngstedt, 2005) consistently show that: (1) when asked an open ended question about behaviors that promote better sleep, exercise is listed as the most important; and (2) based on self-report data, people who exercise more also report having better quality sleep and reduced daytime sleepiness – when compared with individuals who are more sedentary. Exercise effects on sleep quality might be explained by the chronobiotic properties of exercise (i.e., the ability to synchronize circadian rhythms of physiology and behavior). The chronobiotic properties of exercise have been well established in various animal models (for reviews, see Mrosovsky, 1996; Hastings et al., 1998), however, the science is less definitive in humans (reviewed in Mistlberger and Skene, 2004, 2005).

As such, recent research efforts have increased focus on PA and improved circadian regulation of sleep–wake rhythms as a means to promote health and cognitive function in older adults (for relevant reviews, see Colcombe and Kramer, 2003; Landry and Liu-Ambrose, 2014). However, PA and sleep quality are complex constructs to evaluate empirically. Thus, we suggest the validity of future research efforts depends greatly on the methods used to measure parameters of PA and sleep quality.

Recent technological advances have led to the development of battery powered, long-life, light-weight, non-invasive, wrist-worn accelerometers that measure tri-axial movement (i.e., actigraphy). Wrist-worn accelerometers (WWAs) have been validated for measurement of sleep parameters by comparison with polysomnography (PSG; the gold standard objective measure of sleep; Littner et al., 2003a); and thus, actigraphy is currently accepted as a valid, practical alternative to PSG, allowing for long-term continuous sleep assessments at home (Ancoli-Israel et al., 2003; Littner et al., 2003b). Importantly, objective measures (i.e., actigraphy using WWA) survey different aspects of sleep quality – when compared with commonly used subjective measures such as the Pittsburgh Sleep Quality Index (PSQI; Buysse et al., 1989) and the Consensus Sleep Diary (CSD; Carney et al., 2012).

In a companion study to the current study, we provided evidence showing that PSQI and CSD reported sleep quality yields little to no predictive validity for actigraphic measures of sleep quality for older adults (Landry et al., 2015). We compared subjective (i.e., the PSQI and CSD) and objective measures (i.e., actigraphy using WWA) of sleep quality. For actigraphic measurement of sleep quality, we used the latest WWA – the MotionWatch 8^©^ actigraphy system (MW8; CamNtech, Cambridge, UK), which replaced the discontinued Actiwatch 4 and Actiwatch 7. The companion study’s findings suggested that perceived sleep quality is quite different from objective reality, at least for older adults. We believe these results to be important for researchers aiming to include sleep quality as an outcome measure in their studies. As such, we expect actigraphy will be used more widely in future studies examining PA and sleep quality among older adults.

As with sleep quality, precise measurement of PA is critical to advancing our understanding of PA in both health and disease. The general consensus is that objective measures of PA provide more precise measurements in older adult populations (Warren et al., 2010; Kowalski et al., 2012; Falck et al., 2015). In addition to its utility as an objective measure of sleep quality, the MW8 provides long-term continuous 24/7 recordings of PA. As such, the MW8 is an attractive alternative to previously used measures of PA behavior in older adults. The MW8 provides distinct advantages over other objective measures of PA, such as pedometers and hip-worn accelerometers. First, the MW8 simultaneously provides long-term continuous measures of sleep quality and PA. Second, unlike pedometers and hip-worn accelerometers, the MW8 is waterproof and does not need to be removed during activities such as bathing or swimming. Third, the MW8 is specifically designed for comfortable, continuous wear during the day and at night, which improves wearer compliance and minimizes missing data. Finally, the MW8 might capture light activities better (i.e., household chores), especially those that do not involve the lower extremities.

Clearly, the MW8 is an attractive alternative for researchers examining PA and sleep quality concurrently in older adults. However, there is an important gap in the literature, limiting the MW8’s utility as a measure of PA. When used to evaluate sleep quality, the MW8 records data in 60 s epochs. The MW8 provides activity counts – summed in these 60 s epochs – but the problem is interpreting what these counts mean in terms of understanding PA. It makes intuitive sense that higher counts reflect a higher level of activity but what does that mean in terms of recommended levels of daily activity?

The current study aimed to fill this gap in the scientific literature. To achieve this objective we calibrated and validated cut-points for activity counts produced using the MW8 in healthy older adults. Cut-points are used to provide an estimate of activity level and intensity at which the activity is performed (Crouter et al., 2014). Typically, PA is categorized according to one of the following intensities: light, moderate, vigorous, or very-vigorous. Researchers then evaluate the number of minutes a participant engages in PA for a given intensity, over the duration of a specific observation period (e.g., 3 h, whole day; Trost, 2001). In addition, we aimed to determine the reliability of the MW8 for measuring PA in healthy older adults.

## Materials and Methods

### Participants

Community dwelling adults 55+ (*N* = 23) were recruited through advertising in newspapers, pamphlets distributed at local community centers, and word of mouth referrals. Individuals interested in participating in the study were pre-screened for eligibility criteria. We included individuals in the study who: (1) were cleared for exercise using the modified Physical Activity Readiness Questionnaire (Modified PAR-Q; Cardinal et al., 1996; Cardinal and Cardinal, 2000); (2) could walk independently with or without a walking aid; and (3) scored ≥24 on the Mini-Mental State Exam (Folstein et al., 1975). We excluded participants who: (1) had a medical condition precluding them from exercise; (2) had a chronic respiratory condition that could be made worse by exercise; (3) were unable to wear a portable indirect calorimeter during testing; and (4) had limited mobility such that they could not walk 400 m independently. Participants were not excluded based on existence of common age-related co-morbidities such as type-2 diabetes, hypertension, osteoporosis, or history of cancer. However, our inclusion/exclusion criteria were designed to ensure participants were healthy enough to engage in regular moderate to vigorous PA of their own volition. Note: we estimated a correlation between PA measures of *r* ≥ 0.5, requiring a sample size ≥21 to sufficiently power our statistical analyses (G^∗^Power 3.1; Faul et al., 2009).

Ethical approval for this study was obtained from the Vancouver Coastal Health Research Institute and the University of British Columbia’s Clinical Research Ethics Board. All participants provided written informed consent.

### Measures

#### Wrist Worn Accelerometer

We used the MW8 actigraphy system (CamNtech): a light weight, water-proof, tri-axial WWA. In accordance with standard protocol established in previous WWA validation studies, our participants wore the WWA on their non-dominant wrist (Crouter et al., 2014; Johansson et al., 2015). For the purposes of reliability testing, participants wore two WWAs on their non-dominant wrist (Ekblom et al., 2012). Data from the distal WWA (MW8-1) were used to determine activity count cut-points for each PA intensity level (i.e., sedentary, light, and moderate to vigorous). Data from the proximal WWA (MW8-2) were used to test reliability. MW8 data were recorded using 60 s epochs, to match the recommended settings for sleep quality assessments.

#### Indirect Calorimeter

As our criterion measure we used the Cosmed k4b2, a portable indirect calorimeter (Cosmed; Rome, Italy) to determine energy expenditure (EE) during each assessment. Indirect calorimetry is used to measure oxygen uptake (VO2) and production of carbon dioxide (VCO2). Inspired and expired gasses are collected via a breathing mask and then analyzed to determine VO2 and VCO2 volumes. These measurements are used to determine EE in the form of metabolic equivalents (METs) via Schofield-equations (Schofield, 1985); which are then used to classify an activity based on the METs it requires. In accordance with established guidelines (Pate et al., 2008), activities were classified as follows: (1) sedentary (<1.5 METs); (2) light (1.5–3.0 METs), or moderate to vigorous physical activity (MVPA; >3.0 METs).

#### Demographics

Participants were surveyed via questionnaire for their age, sex, race, and ethnicity; as well as any current or previous health conditions such as diabetes, hypertension, osteoporosis, arthritis, and cancer. In addition, participants reported if they used a cane or other ambulatory device. Finally, height and body weight (as measured by a calibrated stadiometer and electronic scale, respectively) were used to determine each participant’s body mass index (BMI; kg/m2).

### Measurement Protocol

The Cosmed gas analyzers were calibrated and verified with known gasses immediately before and after each test. Following calibration completion, the Cosmed was fitted for comfort to each participant. The Cosmed was worn concurrently with both WWAs (i.e., MW8-1 and MW8-2). To improve the accuracy of indirect calorimetry – in accordance with standard procedures (Bouten et al., 1994; Nichols et al., 2000; Schmitz et al., 2005) – participants were asked to fast 3 h prior to arriving for the measurement session.

We used 60 s epochs for WWA data collection, in accordance with previous validation studies (Berntsen et al., 2010). Measurement of METs was also recorded at 60 s intervals on the indirect calorimeter. Total EE (in kilocalories) during each session was determined using indirect calorimetry. The WWA, METs, and EE data from each session were downloaded into Microsoft Excel for further processing. To ensure consistency across measurement sessions, the same assessor – who was previously trained on all aspects of the measurement protocol – calibrated the Cosmed and conducted the trials for all participants. All instruments were synchronized to the same clock, and time was recorded at the beginning and end of each activity to ensure appropriate data comparisons could be made across recording devices. For all sessions, notes on participant activity during the trial were documented.

The measurement session lasted ∼60 min, during which each participant performed 6 different activities designed to mimic activities of daily living: (1) treadmill walking at four different paces; (2) sitting in a chair; (3) cleaning; (4) resistance training; (5) lying down; and (6) standing. To more closely mimic free-living activity, participants were allowed to move their arms freely during these activities. In order to minimize distraction and standardize the testing procedure the measurement session occurred in a quiet room and participants were not permitted to listen to music. Participants were provided standardized instructions (as per a predetermined script), prior to beginning each activity. As described below, participants performed 10 activities – for 5 min each – in the following order.

#### Treadmill Walking

Participants were asked to walk at four different speeds on the treadmill, with each speed intended to mimic a specific pace: *Leisurely*, *Comfortable*, *Moderate*, and *Brisk*. Participants self-selected the treadmill speed for each pace, as described below. This was done for safety reasons, as well as to adjust for variability in fitness level across participants. Once the treadmill speed had been selected for each pace, the participant walked at this pace for 5 min. The following order was used for each self-selected pace.

##### Leisurely pace

Participants were first instructed to walk at a leisurely pace. This treadmill speed was described as a pace the participant would walk at during a casual walk with a friend. Participants were instructed this pace was supposed to be easy, requiring minimal exertion.

##### Comfortable pace

Participants were instructed to walk at a pace they would use for a little light exercise. This treadmill speed was described as being faster than the previous speed, but requiring limited exertion.

##### Moderate pace

Participants were instructed to walk at a pace they would use for moderate exercise or when completing an errand. Participants were instructed to set the treadmill speed at a quick pace, so they would walk with purpose but not urgency.

##### Brisk pace

This treadmill speed was described as a pace the participant would walk if they were running late or needed to get somewhere as fast as possible. It was intended to be the fastest pace the participant could walk at for 5 min without slowing down.

#### Sitting

Participants were instructed to sit in a chair. No instructions were given as to how they must sit, but they were asked to remain seated for the entire 5 min. Typically, the experimenter and participant engaged in polite conversation during this time.

#### Cleaning

Participants were given a broom and duster and asked to sweep the floor of the surrounding area. Participants were told to sweep the area as they normally would in their own home. The same marked off area was used for sweeping by each participant.

#### Resistance Training

Participants engaged in two resistance training activities that are typical for resistance training programs: Bicep Curls and Squats. Participants completed three sets of 10 repetitions for each exercise. Rest between sets of each exercise was permitted as needed.

##### Bicep curls

Instructions were provided as needed to ensure participants performed all bicep curls using proper technique in a slow and controlled manner while sitting. Five pound dumbbells and ten pound dumbbells were used by women and men, respectively.

##### Squats

Participants performed squats as chair squats. This required participants to sit down in a chair and then stand up to complete one repetition. Participants did not use weights for this task.

#### Lying Down

Participants were instructed to lie down on a bed for a period of 5 min.

#### Standing

Participants were asked to remain standing for 5 min, but were given the option to move around the room freely. No additional instructions were given during this time as this period was intended to mimic standing and free-movement as part of routine daily activity. Following completion of this activity, the trial was terminated.

### Statistical Analysis

#### Primary objective

*To derive MW8 activity count cut-points for healthy older adult PA levels for sedentary, light, moderate-to-vigorous activity:* receiver operator characteristics (ROCs) analyses (Berk, 1976) were performed to determine optimal cut-points for the following PA intensities: sedentary activity (<1.5 METs), light activity (1.5–3.0 METs); MVPA (>3.0 METs; NIH, 2008; Pate et al., 2008). For each ROC curve, METs were coded as 0 or 1 according to the cut-point being established. For example, when the sedentary cut-point was being established, a “1” was assigned to all minutes wherein a participant was reported in sedentary activity via indirect calorimetry and a “0” was assigned to all minutes wherein a participant was not reported in sedentary activity. The area under the ROC curve was calculated for PA intensity for both sedentary and MVPA.

Cut-points were established for sedentary activity using the point closest to (0, 1) on the ROC curve (i.e., *d*^2^). This method of using the lowest *d*^2^ is considered one of the two best methods for creating cut-points (Akobeng, 2007). Cut-points for MVPA were established using the lowest *d*^2^ given a false-positive-ratio <0.10. We derived cut-points using this approach to prevent overestimation and provide a more conservative estimate of MVPA. The cut-point for light PA was determined as the activity level between the boundaries for sedentary activity and MVPA.

*Sensitivity*, *Specificity*, *Accuracy*, *Positive Predictive Value*, and *Negative Predictive Value* were calculated for both the sedentary activity and MVPA cut-points. *Sensitivity* is the percentage of epochs correctly identified as being engaged at the activity level examined (e.g., percent of actual MVPA epochs correctly identified as an epoch of MVPA). *Specificity* is the percentage of epochs correctly identified as not being engaged at the activity level examined (e.g., percent of actual non-MVPA epochs correctly identified as not being an epoch of MVPA). *Accuracy* is defined as the percent of correct decisions made using the derived cut-point (e.g., percent correctly identified as MVPA and percent correctly identified as non-MVPA). *Positive Predictive Value* is the probability of correctly identifying an epoch as being engaged at that activity level, given that it truly is an epoch of that activity level (e.g., probability of correctly identifying an epoch of MVPA as MVPA). *Negative Predictive Value* is the probability of correctly identifying an epoch as not being engaged at that activity level, given that it truly is not an epoch of that activity level (e.g., the probability of correctly identifying an epoch of sedentary activity as not being MVPA).

#### Secondary objective

*To test reliability of the MW8:* Using SPSS version 22 (IBM Corporation 2013), data for each WWA (i.e., MW8-1 and MW8-2) were summarized and compared via interclass correlations (ICC) to assess average agreement for both WWA over different levels of activity. A higher ICC is indicative of greater inter-rater reliability. In addition, Bland–Altman plots were generated to determine agreement for both WWA across different levels of activity (Bland and Altman, 1986).

## Results

### Participant Characteristics

**Table 1** describes characteristics of our study participants. The average age was 69.96 years (SD = 6.57). Participants were slightly overweight (BMI: average = 26.64 ± 5.22 kg/m2) as per current guidelines (Flegal et al., 2012) and were predominantly Caucasian (91.30%) and female (69.60%). In addition, participants had few self-reported comorbidities. Osteoporosis was the most common physical ailment reported (30.60%), 4.30% of participants used a cane or other ambulatory device.

**Table 1:** Participant characteristics (*N* = 23).

Participant characteristic	Mean (SD) or %
Age	69.96 (6.57)
BMI	26.64 (5.22)
Females	69.60%
Race	
Caucasian	91.30%
Asian	4.30%
Other	4.30%
Diabetic	4.30%
Cancer	4.30%
Osteoporosis	30.40%
Cane use	4.30%

### Reliability of WWA

**FIGURE 1:**
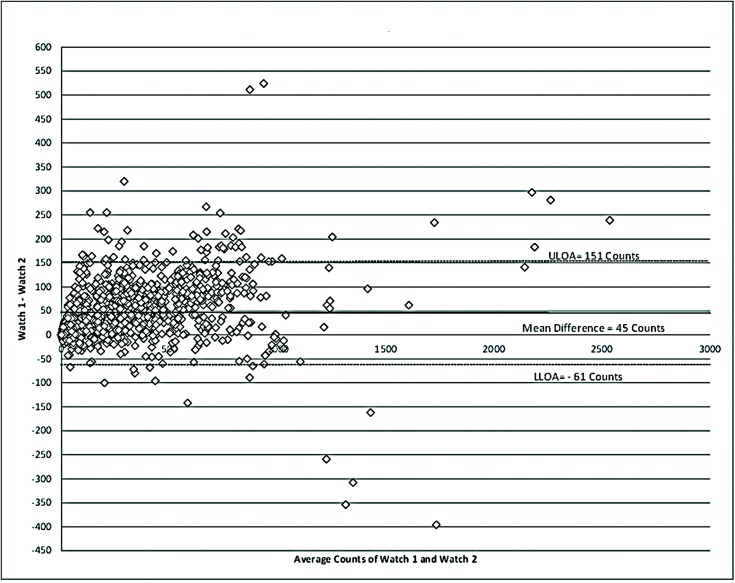
**Bland–Altman for reliability of the MotionWatch**. Differences between counts reported by the distal watch (Watch 1) and proximal watch (Watch 2) are reported. A higher placement of the WWA on the wrist appears to be associated with a slight under-estimation of movement frequency. ULOA, upper limits of agreement; LLOA, lower limits of agreement.

**Table 2** describes the reliability of the WWA during the measurement period. A total of 1217 epochs were captured by each WWA. Mean counts per minute (CPM) for MW8-1 was 321.44 (SD = 316.52 CPM), while MW8-2 had a mean CPM of 276.84 (SD = 295.72 CPM). The average difference between WWA was 45 CPM as shown in **Figure 1**.

**Table 2:** Reliability of the WWA.

Watch	*N*	Mean CPM (SD)	Pearson correlation	ICC (95% CI)
Watch 1	1217	321.44 (316.52)	0.981^∗^	0.979 (0.977, 0.981)^∗^
Watch 2	1217	276.84 (295.72)	–	–

*^∗^p < 0.01.*

The CPM measured for MW8-1 and MW8-2 were strongly correlated (*r* = 0.981; *p* < 0.01). Moreover, the ICC assessing consistency of the measures provided for each WWA also showed very strong agreement of ICC = 0.979 (95% CI: 0.977, 0.981).

### ROC Curve Analysis

**FIGURE 2:**
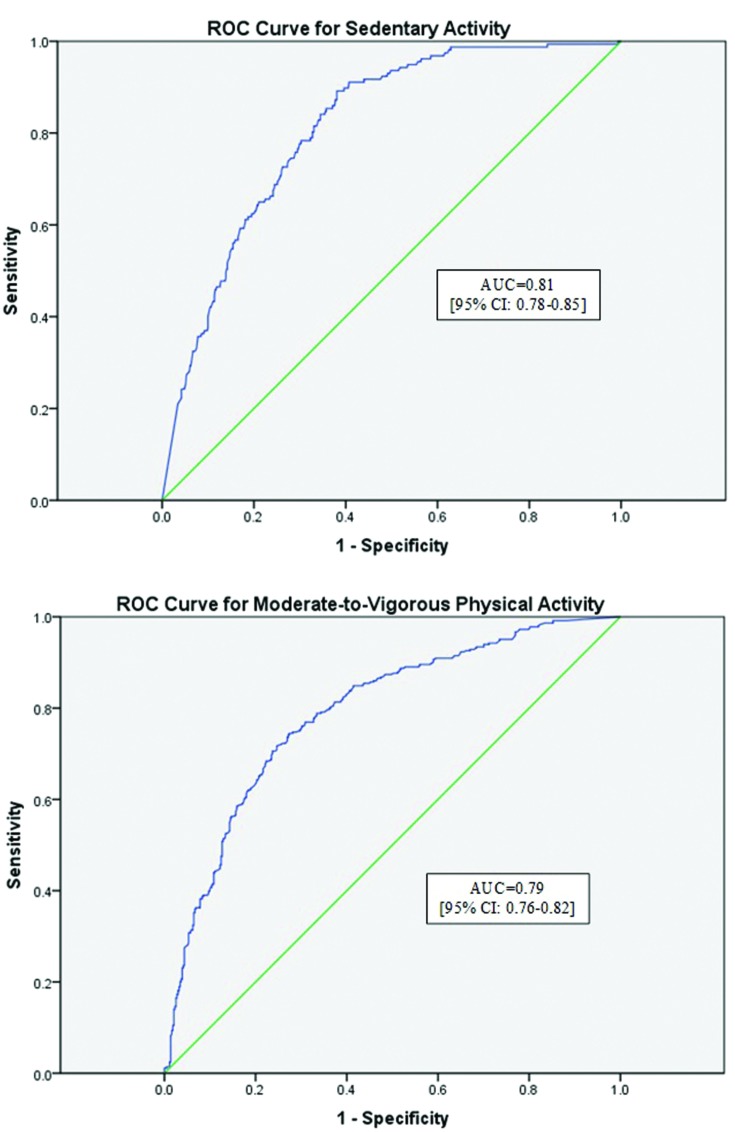
**Receiver operator characteristic (ROC) curves for sedentary activity and moderate-to-vigorous physical activity**. ROC curves for both sedentary activity (Sensitivity = 0.78; Specificity = 0.70) and moderate-to-vigorous activity (Sensitivity = 0.34; Specificity = 0.90) are reported. Moderate agreement was found for the WWA for both activity intensities. AUC, area under the curve.

**Figure 2** describes the ROC curves for sedentary activity and MVPA, respectively. The area under the curve (AUC) for the sedentary ROC curve was deemed moderately accurate at 0.81 (95% CI: 0.78, 0.85), in accordance with previous recommendations for these analyses (Akobeng, 2007). The AUC for MVPA was also moderately accurate at 0.79 (95% CI: 0.76, 0.82).

### Determining Optimal Cut-points

**Table 3** describes the optimum cut-points for sedentary activity and MVPA with light activity being between these boundaries. The cut-point for sedentary behavior determined using the lowest *d*^2^ was ≤178.50 CPM (*d*^2^ = 0.14). Using this cut-point yielded a sensitivity of 78% and specificity of 70%, with an accuracy of 71%. This sedentary activity cut-point had a positive predictive value of 39% and a negative predictive value of 93%. After adjustments to target a false-positive ratio of ≤0.10, the derived cut-point for MVPA was ≥562.50 CPM (*d*^2^ = 0.37). This MVPA cut-point yielded a sensitivity of 40% and specificity of 90%, with an accuracy of 69%. This MVPA cut-point had a positive predictive value of 77% and a negative predictive value of 60%.

**Table 3:** Cut-points for Sedentary and Moderate-Vigorous Activity.

Activity Level	Cut-point	Sensitivity	Specificity	Accuracy	*d*^2^	Positive predictive value	Negative predictive value
Sedentary	≤178.50	0.78	0.70	0.70	0.22	0.39	0.93
MVPA^∗^	≥562.50	0.34	0.90	0.67	0.37	0.77	0.60
Light^∗∗^	178.50–562.50	–	–	–	–	–	–

*^∗^Cut-point determined for lowest d^2^ given specificity ≥ 0.90. ^∗∗^Cut-points for light activity are the boundaries for sedentary activity and MVPA*.

## Discussion

Most objectively measured studies of PA in older adults have used either a pedometer or hip-accelerometer to measure PA (Murphy, 2009). Pedometers are well established tools for measuring step-counts and have been validated for the prediction of EE (Crouter et al., 2003). Hip-accelerometers have also been validated in various populations (Vooijs et al., 2014; Lee et al., 2015), including older adults (Copeland and Esliger, 2009; Taylor et al., 2014).

However, there are important limitations to consider when using hip accelerometers in interventions targeting older adults. First, few cut-points have been calibrated specifically for older adults. Importantly, Gorman et al. (2014) reviewed current studies using hip-accelerometers with older adults, and found the most commonly used cut-points had not been calibrated for older adults, but rather for young adults. This practice of using tools that have not been specifically calibrated for the population in question likely results in misleading findings (Falck et al., 2015). Second, a lack of wearer compliance can be an issue for hip-accelerometers – resulting in missing-data – which can also impact findings. And finally, hip accelerometers – and most pedometers – have not been designed for continuous wear 24/7.

By contrast, the MW8 was designed for long-term, continuous wear to record activity during the day and at night. The MW8 is the latest WWA, replacing the Actiwatch 4 and Actiwatch 7, which were discontinued. Importantly, the MW8 is water-resistant allowing for continuous wear, even during bathing, showers, and while swimming – providing an advantage over most pedometers and hip-accelerometers. Furthermore, the MW8 provides for both PA and sleep quality measurements in one device.

To our knowledge, we provide the first validation of PA cut-points for the MW8, specifically for use with healthy older adults 55+. Furthermore, our findings suggest that in addition to its previously validated use as a measure of sleep quality (Elbaz et al., 2012; Morgan et al., 2012) the MW8 is a reliable tool for measuring PA in healthy older adults. Considering the increasing number of studies testing interventions targeting PA and sleep quality and cognitive function in older adults (e.g., Ko and Youn, 2011; Nguyen and Kruse, 2012; Schega et al., 2013; Figueiro et al., 2014; Pa et al., 2014; Richter et al., 2014; Cordi et al., 2015), and given the relationship between exercise and sleep (Driver and Taylor, 2000; Youngstedt, 2005), our PA cut-points validate a new level of analysis when using the MW8, filling an important gap in the literature.

### Limitations

Our cut-points may be limited by inter-individual differences in physiological stress across participants. Numerous types of stressors including severe illnesses can affect EE (for review see Preiser et al., 2014); however, our sample was generally healthy – as evidenced by limited co-morbidities – such that minimal differences in basal metabolic rate would be expected. We acknowledge the MW8 activity count cut-points defined in the current study lack the accuracy previously reported for the GENEA accelerometer (Esliger et al., 2011). However, the MW8’s utility as a valid objective measure of both PA and sleep quality, make it an attractive tool for researchers working with older adults. We expect the MW8 to be used frequently in future studies, and thus, our cut-points should provide for increased depth of analysis in these studies.

Generalizability of our findings is limited to MW8 use in healthy older adults. Older adults who are frail or confined to a wheelchair will likely have different PA patterns, requiring a unique set of PA cut-points. Furthermore, specific older adult populations such as those who suffer from chronic stroke would also require a unique set of PA cut-points.

## Conflict of Interest Statement

The authors declare that the research was conducted in the absence of any commercial or financial relationships that could be construed as a potential conflict of interest.
