# Supplementary material for: Measuring physical activity in older adults: calibrating cut-points for the MotionWatch 8©
Source: Front Aging Neurosci. 2015 Aug 25;7:165. doi: 10.3389/fnagi.2015.00165 (PMC4548198; doi:10.3389/fnagi.2015.00165)
Supplement: Supplementary file 1 [file Table_1.DOCX]

Supplementary Table 1: Characteristics of Each Potential Cut-point for Sedentary Activity

**Note: Each cut-point for sedentary activity is composed of all counts less than or equal to the given value*

| Cut-point* | Sensitivity | 1 - Specificity | Positive  Predictive  Value | Negative Predictive Value | Accuracy | d^2^ |
| --- | --- | --- | --- | --- | --- | --- |
| 1.0 | 0.000 | 0.000 | 0.000000 | 0.803258 | 0.803258 | 1.000000 |
| 2.0 | 0.210 | 0.034 | 0.602038 | 0.833122 | 0.817263 | 0.625256 |
| 4.5 | 0.223 | 0.042 | 0.565304 | 0.834269 | 0.813395 | 0.605493 |
| 5.5 | 0.242 | 0.042 | 0.585279 | 0.837664 | 0.817133 | 0.576328 |
| 6.5 | 0.242 | 0.044 | 0.573945 | 0.837380 | 0.815526 | 0.576500 |
| 7.5 | 0.242 | 0.045 | 0.568441 | 0.837237 | 0.814723 | 0.576589 |
| 8.5 | 0.242 | 0.048 | 0.552544 | 0.836808 | 0.812313 | 0.576868 |
| 9.5 | 0.248 | 0.050 | 0.548503 | 0.837604 | 0.811887 | 0.568004 |
| 10.5 | 0.255 | 0.051 | 0.550491 | 0.838730 | 0.812461 | 0.557626 |
| 12.0 | 0.274 | 0.053 | 0.558741 | 0.841913 | 0.814593 | 0.529885 |
| 13.5 | 0.280 | 0.059 | 0.537546 | 0.842172 | 0.810954 | 0.521881 |
| 14.5 | 0.293 | 0.061 | 0.540540 | 0.844299 | 0.811905 | 0.503570 |
| 15.5 | 0.293 | 0.062 | 0.536499 | 0.844159 | 0.811102 | 0.503693 |
| 16.5 | 0.312 | 0.066 | 0.536576 | 0.847157 | 0.811627 | 0.477700 |
| 17.5 | 0.325 | 0.067 | 0.542981 | 0.849473 | 0.813381 | 0.460114 |
| 18.5 | 0.325 | 0.072 | 0.525073 | 0.848785 | 0.809365 | 0.460809 |
| 19.5 | 0.331 | 0.075 | 0.519452 | 0.849514 | 0.808135 | 0.453186 |
| 21.0 | 0.344 | 0.076 | 0.525758 | 0.851869 | 0.809890 | 0.436112 |
| 22.5 | 0.357 | 0.078 | 0.528530 | 0.854107 | 0.810841 | 0.419533 |
| 23.5 | 0.357 | 0.084 | 0.510032 | 0.853292 | 0.806021 | 0.420505 |
| 24.5 | 0.363 | 0.086 | 0.508317 | 0.854189 | 0.805595 | 0.413165 |
| 25.5 | 0.363 | 0.087 | 0.505428 | 0.854053 | 0.804792 | 0.413338 |
| 26.5 | 0.363 | 0.090 | 0.496952 | 0.853642 | 0.802382 | 0.413869 |
| 27.5 | 0.369 | 0.092 | 0.495556 | 0.854547 | 0.801956 | 0.406625 |
| 29.0 | 0.369 | 0.098 | 0.479772 | 0.853721 | 0.797137 | 0.407765 |
| 30.5 | 0.376 | 0.100 | 0.479420 | 0.854834 | 0.796907 | 0.399376 |
| 31.5 | 0.389 | 0.100 | 0.487909 | 0.857427 | 0.799465 | 0.383321 |
| 32.5 | 0.401 | 0.100 | 0.495502 | 0.859835 | 0.801826 | 0.368801 |
| 33.5 | 0.408 | 0.101 | 0.497341 | 0.861112 | 0.802400 | 0.360665 |
| 34.5 | 0.420 | 0.105 | 0.494878 | 0.863017 | 0.801548 | 0.347425 |
| 35.5 | 0.420 | 0.106 | 0.492509 | 0.862885 | 0.800744 | 0.347636 |
| 36.5 | 0.433 | 0.111 | 0.488608 | 0.864891 | 0.799286 | 0.333810 |
| 37.5 | 0.433 | 0.114 | 0.481947 | 0.864496 | 0.796876 | 0.334485 |
| 38.5 | 0.446 | 0.114 | 0.489336 | 0.867190 | 0.799434 | 0.319912 |
| 39.5 | 0.459 | 0.115 | 0.494334 | 0.869773 | 0.801188 | 0.305906 |
| 41.0 | 0.459 | 0.117 | 0.490024 | 0.869516 | 0.799581 | 0.306370 |
| 42.5 | 0.465 | 0.119 | 0.489034 | 0.870521 | 0.799155 | 0.300386 |
| 44.0 | 0.465 | 0.122 | 0.482815 | 0.870136 | 0.796746 | 0.301109 |
| 45.5 | 0.465 | 0.123 | 0.480777 | 0.870007 | 0.795942 | 0.301354 |
| 46.5 | 0.465 | 0.126 | 0.474764 | 0.869619 | 0.793533 | 0.302101 |
| 47.5 | 0.471 | 0.126 | 0.477962 | 0.870893 | 0.794713 | 0.295717 |
| 48.5 | 0.478 | 0.128 | 0.477714 | 0.872128 | 0.794484 | 0.288868 |
| 49.5 | 0.478 | 0.131 | 0.471937 | 0.871743 | 0.792074 | 0.289645 |

Supplementary Table 1 (Continued)

| Cut-point* | Sensitivity | 1 - Specificity | Positive  Predictive  Value | Negative Predictive Value | Accuracy | d^2^ |
| --- | --- | --- | --- | --- | --- | --- |
| 51.5 | 0.478 | 0.136 | 0.462613 | 0.871097 | 0.788058 | 0.290980 |
| 52.5 | 0.478 | 0.137 | 0.460792 | 0.870966 | 0.787254 | 0.291253 |
| 54.0 | 0.484 | 0.139 | 0.460291 | 0.872001 | 0.786828 | 0.285577 |
| 55.5 | 0.490 | 0.139 | 0.463353 | 0.873301 | 0.788009 | 0.279421 |
| 56.5 | 0.497 | 0.139 | 0.466882 | 0.874822 | 0.789386 | 0.272330 |
| 57.5 | 0.503 | 0.140 | 0.468084 | 0.876005 | 0.789763 | 0.266609 |
| 58.5 | 0.503 | 0.142 | 0.464554 | 0.875751 | 0.788157 | 0.267173 |
| 59.5 | 0.510 | 0.142 | 0.467994 | 0.877287 | 0.789534 | 0.260264 |
| 60.5 | 0.516 | 0.142 | 0.470907 | 0.878607 | 0.790714 | 0.254420 |
| 61.5 | 0.522 | 0.144 | 0.470303 | 0.879684 | 0.790288 | 0.249220 |
| 62.5 | 0.529 | 0.147 | 0.468485 | 0.880869 | 0.789256 | 0.243450 |
| 63.5 | 0.541 | 0.148 | 0.472384 | 0.883430 | 0.790813 | 0.232585 |
| 64.5 | 0.548 | 0.151 | 0.470587 | 0.884644 | 0.789781 | 0.227105 |
| 65.5 | 0.548 | 0.154 | 0.465689 | 0.884282 | 0.787371 | 0.228020 |
| 66.5 | 0.561 | 0.154 | 0.471527 | 0.887235 | 0.789929 | 0.216437 |
| 68.0 | 0.561 | 0.156 | 0.468313 | 0.886998 | 0.788322 | 0.217057 |
| 70.0 | 0.561 | 0.158 | 0.465142 | 0.886760 | 0.786716 | 0.217685 |
| 71.5 | 0.567 | 0.159 | 0.466219 | 0.888016 | 0.787093 | 0.212770 |
| 72.5 | 0.567 | 0.162 | 0.461571 | 0.887661 | 0.784683 | 0.213733 |
| 73.5 | 0.567 | 0.165 | 0.457014 | 0.887302 | 0.782273 | 0.214714 |
| 74.5 | 0.573 | 0.165 | 0.459627 | 0.888690 | 0.783454 | 0.209554 |
| 75.5 | 0.586 | 0.168 | 0.460724 | 0.891364 | 0.783602 | 0.199620 |
| 76.5 | 0.592 | 0.170 | 0.460315 | 0.892539 | 0.783175 | 0.195364 |
| 77.5 | 0.592 | 0.178 | 0.448914 | 0.891607 | 0.776749 | 0.198148 |
| 79.0 | 0.599 | 0.179 | 0.450436 | 0.893152 | 0.777323 | 0.192842 |
| 80.5 | 0.605 | 0.181 | 0.450153 | 0.894352 | 0.776897 | 0.188786 |
| 81.5 | 0.611 | 0.181 | 0.452597 | 0.895789 | 0.778078 | 0.184082 |
| 83.5 | 0.611 | 0.183 | 0.449876 | 0.895561 | 0.776471 | 0.184810 |
| 86.0 | 0.611 | 0.184 | 0.448527 | 0.895446 | 0.775668 | 0.185177 |
| 87.5 | 0.611 | 0.187 | 0.444530 | 0.895101 | 0.773258 | 0.186290 |
| 89.0 | 0.618 | 0.187 | 0.447345 | 0.896794 | 0.774635 | 0.180893 |
| 90.5 | 0.618 | 0.190 | 0.443414 | 0.896451 | 0.772226 | 0.182024 |
| 91.5 | 0.618 | 0.193 | 0.439551 | 0.896106 | 0.769816 | 0.183173 |
| 92.5 | 0.618 | 0.195 | 0.437013 | 0.895875 | 0.768209 | 0.183949 |
| 94.0 | 0.624 | 0.195 | 0.439391 | 0.897342 | 0.769390 | 0.179401 |
| 95.5 | 0.624 | 0.197 | 0.436879 | 0.897113 | 0.767783 | 0.180185 |
| 97.0 | 0.624 | 0.198 | 0.435634 | 0.896998 | 0.766980 | 0.180580 |
| 99.0 | 0.624 | 0.200 | 0.433165 | 0.896767 | 0.765373 | 0.181376 |
| 101.0 | 0.631 | 0.203 | 0.432248 | 0.898151 | 0.764341 | 0.177370 |
| 102.5 | 0.631 | 0.204 | 0.431043 | 0.898036 | 0.763538 | 0.177777 |
| 103.5 | 0.637 | 0.206 | 0.430971 | 0.899299 | 0.763112 | 0.174205 |
| 104.5 | 0.643 | 0.207 | 0.432083 | 0.900686 | 0.763489 | 0.170298 |
| 107.0 | 0.643 | 0.209 | 0.429725 | 0.900460 | 0.761882 | 0.171130 |
| 109.5 | 0.650 | 0.211 | 0.430045 | 0.901997 | 0.761653 | 0.167021 |
| 111.0 | 0.650 | 0.214 | 0.426588 | 0.901660 | 0.759243 | 0.168296 |
| 112.5 | 0.650 | 0.215 | 0.425448 | 0.901547 | 0.758440 | 0.168725 |

Supplementary Table 1 (Continued)

| Cut-point* | Sensitivity | 1 - Specificity | Positive  Predictive  Value | Negative Predictive Value | Accuracy | d^2^ |
| --- | --- | --- | --- | --- | --- | --- |
| 113.5 | 0.650 | 0.218 | 0.422064 | 0.901207 | 0.756030 | 0.170024 |
| 115.0 | 0.650 | 0.223 | 0.416543 | 0.900634 | 0.752014 | 0.172229 |
| 116.5 | 0.656 | 0.225 | 0.416606 | 0.901943 | 0.751588 | 0.168961 |
| 117.5 | 0.656 | 0.229 | 0.412329 | 0.901485 | 0.748375 | 0.170777 |
| 118.5 | 0.656 | 0.231 | 0.410224 | 0.901254 | 0.746768 | 0.171697 |
| 119.5 | 0.656 | 0.232 | 0.409179 | 0.901138 | 0.745965 | 0.172160 |
| 121.0 | 0.656 | 0.234 | 0.407106 | 0.900905 | 0.744358 | 0.173092 |
| 123.5 | 0.662 | 0.236 | 0.407249 | 0.902235 | 0.743932 | 0.169940 |
| 125.5 | 0.662 | 0.237 | 0.406229 | 0.902119 | 0.743129 | 0.170413 |
| 126.5 | 0.662 | 0.239 | 0.404203 | 0.901887 | 0.741523 | 0.171365 |
| 127.5 | 0.662 | 0.240 | 0.403198 | 0.901771 | 0.740719 | 0.171844 |
| 128.5 | 0.662 | 0.242 | 0.401203 | 0.901537 | 0.739113 | 0.172808 |
| 129.5 | 0.675 | 0.242 | 0.405884 | 0.904964 | 0.741670 | 0.164189 |
| 131.5 | 0.682 | 0.245 | 0.405401 | 0.906485 | 0.740638 | 0.161149 |
| 134.5 | 0.688 | 0.245 | 0.407514 | 0.908087 | 0.741818 | 0.157369 |
| 136.5 | 0.688 | 0.250 | 0.402645 | 0.907531 | 0.737802 | 0.159844 |
| 137.5 | 0.688 | 0.251 | 0.401685 | 0.907419 | 0.736999 | 0.160345 |
| 139.0 | 0.694 | 0.251 | 0.403774 | 0.909037 | 0.738179 | 0.156637 |
| 140.5 | 0.701 | 0.256 | 0.401444 | 0.910388 | 0.735540 | 0.154937 |
| 142.0 | 0.707 | 0.257 | 0.402555 | 0.911920 | 0.735917 | 0.151898 |
| 143.5 | 0.707 | 0.259 | 0.400692 | 0.911703 | 0.734311 | 0.152930 |
| 144.5 | 0.720 | 0.261 | 0.403223 | 0.915079 | 0.735262 | 0.146521 |
| 145.5 | 0.726 | 0.262 | 0.404300 | 0.916644 | 0.735639 | 0.143720 |
| 146.5 | 0.726 | 0.264 | 0.402470 | 0.916436 | 0.734033 | 0.144772 |
| 147.5 | 0.726 | 0.265 | 0.401561 | 0.916332 | 0.733229 | 0.145301 |
| 149.0 | 0.726 | 0.267 | 0.399756 | 0.916123 | 0.731623 | 0.146365 |
| 150.5 | 0.726 | 0.268 | 0.398859 | 0.916018 | 0.730820 | 0.146900 |
| 151.5 | 0.726 | 0.270 | 0.397078 | 0.915807 | 0.729213 | 0.147976 |
| 152.5 | 0.726 | 0.271 | 0.396193 | 0.915702 | 0.728410 | 0.148517 |
| 153.5 | 0.726 | 0.273 | 0.394436 | 0.915489 | 0.726803 | 0.149605 |
| 154.5 | 0.732 | 0.273 | 0.396403 | 0.917187 | 0.727984 | 0.146353 |
| 155.5 | 0.739 | 0.275 | 0.396934 | 0.918970 | 0.727754 | 0.143746 |
| 157.5 | 0.745 | 0.281 | 0.393708 | 0.920076 | 0.724115 | 0.143986 |
| 159.5 | 0.745 | 0.284 | 0.391176 | 0.919768 | 0.721706 | 0.145681 |
| 160.5 | 0.745 | 0.285 | 0.390339 | 0.919665 | 0.720902 | 0.146250 |
| 161.5 | 0.745 | 0.287 | 0.388676 | 0.919458 | 0.719296 | 0.147394 |
| 163.0 | 0.752 | 0.289 | 0.389248 | 0.921292 | 0.719066 | 0.145025 |
| 164.5 | 0.758 | 0.290 | 0.390317 | 0.922949 | 0.719444 | 0.142664 |
| 165.5 | 0.758 | 0.292 | 0.388682 | 0.922748 | 0.717837 | 0.143828 |
| 166.5 | 0.758 | 0.293 | 0.387870 | 0.922648 | 0.717034 | 0.144413 |
| 167.5 | 0.758 | 0.295 | 0.386256 | 0.922445 | 0.715427 | 0.145589 |
| 168.5 | 0.764 | 0.295 | 0.388127 | 0.924222 | 0.716608 | 0.142721 |
| 170.5 | 0.764 | 0.296 | 0.387324 | 0.924123 | 0.715805 | 0.143312 |
| 172.5 | 0.777 | 0.300 | 0.388143 | 0.927620 | 0.715149 | 0.139729 |
| 174.0 | 0.777 | 0.301 | 0.387353 | 0.927524 | 0.714346 | 0.140330 |
| 176.5 | 0.777 | 0.303 | 0.385782 | 0.927331 | 0.712739 | 0.141538 |

Supplementary Table 1 (Continued)

| Cut-point* | Sensitivity | 1 - Specificity | Positive  Predictive  Value | Negative Predictive Value | Accuracy | d^2^ |
| --- | --- | --- | --- | --- | --- | --- |
| 179.5 | 0.783 | 0.304 | 0.386825 | 0.929053 | 0.713117 | 0.139505 |
| 180.5 | 0.783 | 0.307 | 0.384498 | 0.928768 | 0.710707 | 0.141338 |
| 182.0 | 0.783 | 0.312 | 0.380682 | 0.928287 | 0.706690 | 0.144433 |
| 183.5 | 0.783 | 0.314 | 0.379177 | 0.928093 | 0.705084 | 0.145685 |
| 184.5 | 0.783 | 0.318 | 0.376202 | 0.927702 | 0.701871 | 0.148213 |
| 185.5 | 0.783 | 0.320 | 0.374731 | 0.927505 | 0.700264 | 0.149489 |
| 188.0 | 0.783 | 0.321 | 0.374001 | 0.927406 | 0.699461 | 0.150130 |
| 191.0 | 0.783 | 0.323 | 0.372548 | 0.927207 | 0.697855 | 0.151418 |
| 192.5 | 0.790 | 0.323 | 0.374630 | 0.929389 | 0.699232 | 0.148429 |
| 193.5 | 0.790 | 0.326 | 0.372467 | 0.929097 | 0.696822 | 0.150376 |
| 195.0 | 0.790 | 0.328 | 0.371038 | 0.928901 | 0.695216 | 0.151684 |
| 196.5 | 0.803 | 0.328 | 0.374855 | 0.933008 | 0.697773 | 0.146393 |
| 197.5 | 0.803 | 0.329 | 0.374142 | 0.932915 | 0.696970 | 0.147050 |
| 198.5 | 0.803 | 0.331 | 0.372724 | 0.932728 | 0.695363 | 0.148370 |
| 200.5 | 0.809 | 0.331 | 0.374466 | 0.934643 | 0.696544 | 0.146042 |
| 202.5 | 0.815 | 0.331 | 0.376199 | 0.936566 | 0.697724 | 0.143786 |
| 204.5 | 0.815 | 0.335 | 0.373384 | 0.936208 | 0.694511 | 0.146450 |
| 206.5 | 0.815 | 0.337 | 0.371992 | 0.936028 | 0.692905 | 0.147794 |
| 207.5 | 0.828 | 0.340 | 0.373620 | 0.940000 | 0.693053 | 0.145184 |
| 208.5 | 0.828 | 0.342 | 0.372249 | 0.939828 | 0.691446 | 0.146548 |
| 212.0 | 0.828 | 0.343 | 0.371567 | 0.939742 | 0.690643 | 0.147233 |
| 216.0 | 0.828 | 0.345 | 0.370210 | 0.939569 | 0.689036 | 0.148609 |
| 218.0 | 0.841 | 0.345 | 0.373850 | 0.943880 | 0.691594 | 0.144306 |
| 219.5 | 0.841 | 0.348 | 0.371825 | 0.943637 | 0.689184 | 0.146385 |
| 220.5 | 0.841 | 0.353 | 0.368499 | 0.943226 | 0.685168 | 0.149890 |
| 221.5 | 0.841 | 0.354 | 0.367841 | 0.943143 | 0.684365 | 0.150597 |
| 222.5 | 0.847 | 0.356 | 0.368184 | 0.945010 | 0.683939 | 0.150145 |
| 223.5 | 0.847 | 0.357 | 0.367532 | 0.944929 | 0.683135 | 0.150858 |
| 224.5 | 0.854 | 0.357 | 0.369447 | 0.947316 | 0.684513 | 0.148765 |
| 225.5 | 0.854 | 0.360 | 0.367500 | 0.947082 | 0.682103 | 0.150916 |
| 227.0 | 0.854 | 0.362 | 0.366213 | 0.946925 | 0.680496 | 0.152360 |
| 229.0 | 0.854 | 0.363 | 0.365573 | 0.946846 | 0.679693 | 0.153085 |
| 231.0 | 0.854 | 0.365 | 0.364300 | 0.946688 | 0.678086 | 0.154541 |
| 232.5 | 0.854 | 0.370 | 0.361155 | 0.946287 | 0.674070 | 0.158216 |
| 233.5 | 0.854 | 0.371 | 0.360532 | 0.946207 | 0.673267 | 0.158957 |
| 234.5 | 0.860 | 0.371 | 0.362148 | 0.948303 | 0.674447 | 0.157241 |
| 235.5 | 0.860 | 0.373 | 0.360907 | 0.948147 | 0.672841 | 0.158729 |
| 238.0 | 0.860 | 0.376 | 0.359061 | 0.947910 | 0.670431 | 0.160976 |
| 241.0 | 0.873 | 0.378 | 0.361296 | 0.952372 | 0.671382 | 0.159013 |
| 244.5 | 0.873 | 0.379 | 0.360687 | 0.952299 | 0.670579 | 0.159770 |
| 247.5 | 0.873 | 0.381 | 0.359474 | 0.952152 | 0.668972 | 0.161290 |
| 250.5 | 0.885 | 0.381 | 0.362624 | 0.956477 | 0.671333 | 0.158386 |
| 253.5 | 0.892 | 0.381 | 0.364446 | 0.959017 | 0.672711 | 0.156825 |
| 255.0 | 0.892 | 0.382 | 0.363839 | 0.958954 | 0.671907 | 0.157588 |
| 257.0 | 0.892 | 0.387 | 0.360835 | 0.958633 | 0.667891 | 0.161433 |
| 259.0 | 0.892 | 0.390 | 0.359056 | 0.958438 | 0.665481 | 0.163764 |

Supplementary Table 1 (Continued)

| Cut-point* | Sensitivity | 1 - Specificity | Positive  Predictive  Value | Negative Predictive Value | Accuracy | d^2^ |
| --- | --- | --- | --- | --- | --- | --- |
| 263.0 | 0.892 | 0.395 | 0.356129 | 0.958109 | 0.661465 | 0.167689 |
| 264.5 | 0.892 | 0.396 | 0.355550 | 0.958042 | 0.660662 | 0.168480 |
| 266.5 | 0.898 | 0.396 | 0.357087 | 0.960281 | 0.661842 | 0.167220 |
| 268.5 | 0.898 | 0.398 | 0.355932 | 0.960154 | 0.660236 | 0.168808 |
| 269.5 | 0.898 | 0.399 | 0.355357 | 0.960090 | 0.659432 | 0.169605 |
| 270.5 | 0.898 | 0.406 | 0.351383 | 0.959639 | 0.653810 | 0.175240 |
| 272.5 | 0.911 | 0.407 | 0.354103 | 0.964543 | 0.655564 | 0.173570 |
| 275.0 | 0.911 | 0.409 | 0.352982 | 0.964428 | 0.653957 | 0.175202 |
| 276.5 | 0.911 | 0.410 | 0.352425 | 0.964369 | 0.653154 | 0.176021 |
| 278.0 | 0.911 | 0.412 | 0.351315 | 0.964253 | 0.651548 | 0.177665 |
| 279.5 | 0.911 | 0.413 | 0.350763 | 0.964194 | 0.650744 | 0.178490 |
| 280.5 | 0.911 | 0.415 | 0.349663 | 0.964076 | 0.649138 | 0.180146 |
| 281.5 | 0.911 | 0.417 | 0.348571 | 0.963957 | 0.647531 | 0.181810 |
| 283.0 | 0.911 | 0.420 | 0.346945 | 0.963777 | 0.645122 | 0.184321 |
| 285.0 | 0.911 | 0.421 | 0.346406 | 0.963717 | 0.644318 | 0.185162 |
| 286.5 | 0.911 | 0.423 | 0.345334 | 0.963596 | 0.642712 | 0.186850 |
| 288.0 | 0.911 | 0.426 | 0.343738 | 0.963413 | 0.640302 | 0.189397 |
| 289.5 | 0.911 | 0.429 | 0.342157 | 0.963227 | 0.637892 | 0.191962 |
| 290.5 | 0.911 | 0.432 | 0.340590 | 0.963040 | 0.635482 | 0.194545 |
| 291.5 | 0.911 | 0.435 | 0.339037 | 0.962851 | 0.633073 | 0.197146 |
| 292.5 | 0.911 | 0.437 | 0.338010 | 0.962724 | 0.631466 | 0.198890 |
| 296.0 | 0.911 | 0.440 | 0.336481 | 0.962532 | 0.629056 | 0.201521 |
| 299.5 | 0.917 | 0.440 | 0.337948 | 0.964970 | 0.630237 | 0.200489 |
| 301.5 | 0.917 | 0.446 | 0.334925 | 0.964604 | 0.625417 | 0.205805 |
| 303.5 | 0.917 | 0.448 | 0.333929 | 0.964480 | 0.623811 | 0.207593 |
| 304.5 | 0.917 | 0.449 | 0.333433 | 0.964418 | 0.623008 | 0.208490 |
| 305.5 | 0.917 | 0.451 | 0.332446 | 0.964293 | 0.621401 | 0.210290 |
| 307.0 | 0.917 | 0.454 | 0.330976 | 0.964104 | 0.618991 | 0.213005 |
| 308.5 | 0.917 | 0.456 | 0.330004 | 0.963976 | 0.617385 | 0.214825 |
| 309.5 | 0.917 | 0.457 | 0.329519 | 0.963912 | 0.616581 | 0.215738 |
| 311.5 | 0.917 | 0.459 | 0.328555 | 0.963784 | 0.614975 | 0.217570 |
| 314.5 | 0.917 | 0.460 | 0.328075 | 0.963719 | 0.614172 | 0.218489 |
| 317.0 | 0.917 | 0.462 | 0.327120 | 0.963589 | 0.612565 | 0.220333 |
| 318.5 | 0.917 | 0.465 | 0.325697 | 0.963393 | 0.610155 | 0.223114 |
| 320.5 | 0.917 | 0.466 | 0.325225 | 0.963327 | 0.609352 | 0.224045 |
| 322.5 | 0.917 | 0.470 | 0.323352 | 0.963060 | 0.606139 | 0.227789 |
| 326.0 | 0.917 | 0.471 | 0.322887 | 0.962993 | 0.605336 | 0.228730 |
| 329.5 | 0.917 | 0.473 | 0.321962 | 0.962858 | 0.603729 | 0.230618 |
| 331.0 | 0.917 | 0.476 | 0.320583 | 0.962653 | 0.601320 | 0.233465 |
| 332.5 | 0.924 | 0.479 | 0.320871 | 0.965504 | 0.600287 | 0.235217 |
| 333.5 | 0.924 | 0.480 | 0.320417 | 0.965440 | 0.599484 | 0.236176 |
| 334.5 | 0.924 | 0.484 | 0.318612 | 0.965181 | 0.596271 | 0.240032 |
| 337.5 | 0.924 | 0.487 | 0.317272 | 0.964985 | 0.593861 | 0.242945 |
| 340.5 | 0.924 | 0.490 | 0.315944 | 0.964786 | 0.591451 | 0.245876 |
| 343.5 | 0.930 | 0.491 | 0.316903 | 0.967414 | 0.591828 | 0.245981 |
| 346.5 | 0.930 | 0.493 | 0.316023 | 0.967289 | 0.590222 | 0.247949 |

Supplementary Table 1 (Continued)

| Cut-point* | Sensitivity | 1 - Specificity | Positive  Predictive  Value | Negative Predictive Value | Accuracy | d^2^ |
| --- | --- | --- | --- | --- | --- | --- |
| 350.0 | 0.930 | 0.496 | 0.314713 | 0.967101 | 0.587812 | 0.250916 |
| 354.0 | 0.936 | 0.498 | 0.315233 | 0.969719 | 0.587386 | 0.252100 |
| 355.5 | 0.936 | 0.499 | 0.314800 | 0.969661 | 0.586583 | 0.253097 |
| 357.5 | 0.936 | 0.501 | 0.313938 | 0.969543 | 0.584976 | 0.255097 |
| 359.5 | 0.936 | 0.502 | 0.313508 | 0.969484 | 0.584173 | 0.256100 |
| 362.0 | 0.936 | 0.504 | 0.312653 | 0.969364 | 0.582566 | 0.258112 |
| 365.5 | 0.936 | 0.505 | 0.312227 | 0.969304 | 0.581763 | 0.259121 |
| 367.5 | 0.936 | 0.507 | 0.311379 | 0.969184 | 0.580157 | 0.261145 |
| 368.5 | 0.936 | 0.512 | 0.309279 | 0.968878 | 0.576140 | 0.266240 |
| 369.5 | 0.936 | 0.513 | 0.308862 | 0.968816 | 0.575337 | 0.267265 |
| 371.5 | 0.936 | 0.515 | 0.308032 | 0.968691 | 0.573731 | 0.269321 |
| 373.5 | 0.943 | 0.518 | 0.308382 | 0.971851 | 0.572698 | 0.271573 |
| 374.5 | 0.943 | 0.524 | 0.305932 | 0.971506 | 0.567878 | 0.277825 |
| 375.5 | 0.943 | 0.532 | 0.302724 | 0.971033 | 0.561452 | 0.286273 |
| 379.5 | 0.943 | 0.534 | 0.301932 | 0.970912 | 0.559846 | 0.288405 |
| 385.0 | 0.949 | 0.535 | 0.302876 | 0.973839 | 0.560223 | 0.288826 |
| 389.0 | 0.949 | 0.537 | 0.302088 | 0.973729 | 0.558617 | 0.290970 |
| 393.0 | 0.949 | 0.538 | 0.301696 | 0.973674 | 0.557813 | 0.292045 |
| 395.5 | 0.949 | 0.543 | 0.299751 | 0.973394 | 0.553797 | 0.297450 |
| 397.0 | 0.949 | 0.544 | 0.299365 | 0.973337 | 0.552994 | 0.298537 |
| 399.0 | 0.949 | 0.546 | 0.298596 | 0.973223 | 0.551387 | 0.300717 |
| 401.0 | 0.949 | 0.548 | 0.297831 | 0.973107 | 0.549781 | 0.302905 |
| 404.0 | 0.949 | 0.549 | 0.297449 | 0.973049 | 0.548977 | 0.304002 |
| 408.0 | 0.949 | 0.551 | 0.296690 | 0.972932 | 0.547371 | 0.306202 |
| 411.0 | 0.949 | 0.552 | 0.296312 | 0.972874 | 0.546568 | 0.307305 |
| 413.0 | 0.949 | 0.554 | 0.295558 | 0.972755 | 0.544961 | 0.309517 |
| 414.5 | 0.955 | 0.557 | 0.295746 | 0.975724 | 0.543732 | 0.312274 |
| 416.0 | 0.955 | 0.559 | 0.295000 | 0.975617 | 0.542125 | 0.314506 |
| 417.5 | 0.955 | 0.560 | 0.294629 | 0.975563 | 0.541322 | 0.315625 |
| 420.0 | 0.955 | 0.563 | 0.293519 | 0.975399 | 0.538912 | 0.318994 |
| 422.5 | 0.962 | 0.565 | 0.294299 | 0.979052 | 0.538683 | 0.320669 |
| 424.0 | 0.962 | 0.568 | 0.293200 | 0.978910 | 0.536273 | 0.324068 |
| 426.0 | 0.962 | 0.569 | 0.292836 | 0.978862 | 0.535470 | 0.325205 |
| 427.5 | 0.962 | 0.573 | 0.291387 | 0.978668 | 0.532257 | 0.329773 |
| 431.0 | 0.962 | 0.577 | 0.289953 | 0.978471 | 0.529044 | 0.334373 |
| 435.0 | 0.962 | 0.579 | 0.289241 | 0.978371 | 0.527437 | 0.336685 |
| 436.5 | 0.962 | 0.580 | 0.288887 | 0.978320 | 0.526634 | 0.337844 |
| 438.0 | 0.962 | 0.582 | 0.288180 | 0.978219 | 0.525028 | 0.340168 |
| 439.5 | 0.962 | 0.585 | 0.287127 | 0.978065 | 0.522618 | 0.343669 |
| 440.5 | 0.968 | 0.585 | 0.288401 | 0.981464 | 0.523798 | 0.343249 |
| 441.5 | 0.968 | 0.587 | 0.287701 | 0.981376 | 0.522192 | 0.345593 |
| 443.5 | 0.968 | 0.588 | 0.287352 | 0.981331 | 0.521388 | 0.346768 |
| 445.5 | 0.968 | 0.590 | 0.286657 | 0.981242 | 0.519782 | 0.349124 |
| 448.0 | 0.968 | 0.591 | 0.286311 | 0.981197 | 0.518979 | 0.350305 |
| 450.5 | 0.968 | 0.594 | 0.285278 | 0.981061 | 0.516569 | 0.353860 |
| 451.5 | 0.968 | 0.599 | 0.283572 | 0.980829 | 0.512553 | 0.359825 |

Supplementary Table 1 (Continued)

| Cut-point* | Sensitivity | 1 - Specificity | Positive  Predictive  Value | Negative Predictive Value | Accuracy | d^2^ |
| --- | --- | --- | --- | --- | --- | --- |
| 452.5 | 0.968 | 0.601 | 0.282895 | 0.980735 | 0.510946 | 0.362225 |
| 453.5 | 0.968 | 0.602 | 0.282558 | 0.980687 | 0.510143 | 0.363428 |
| 454.5 | 0.968 | 0.604 | 0.281886 | 0.980592 | 0.508536 | 0.365840 |
| 455.5 | 0.968 | 0.607 | 0.280884 | 0.980447 | 0.506127 | 0.369473 |
| 456.5 | 0.968 | 0.608 | 0.280552 | 0.980398 | 0.505323 | 0.370688 |
| 457.5 | 0.968 | 0.610 | 0.279889 | 0.980299 | 0.503717 | 0.373124 |
| 459.0 | 0.968 | 0.613 | 0.278902 | 0.980149 | 0.501307 | 0.376793 |
| 460.5 | 0.975 | 0.616 | 0.279369 | 0.984304 | 0.500274 | 0.380081 |
| 462.0 | 0.975 | 0.618 | 0.278717 | 0.984223 | 0.498668 | 0.382549 |
| 464.5 | 0.975 | 0.619 | 0.278392 | 0.984183 | 0.497865 | 0.383786 |
| 467.0 | 0.975 | 0.622 | 0.277422 | 0.984059 | 0.495455 | 0.387509 |
| 468.5 | 0.981 | 0.624 | 0.278009 | 0.987775 | 0.495029 | 0.389737 |
| 470.0 | 0.981 | 0.626 | 0.277367 | 0.987710 | 0.493422 | 0.392237 |
| 471.5 | 0.981 | 0.627 | 0.277047 | 0.987677 | 0.492619 | 0.393490 |
| 472.5 | 0.981 | 0.629 | 0.276409 | 0.987612 | 0.491013 | 0.396002 |
| 473.5 | 0.987 | 0.629 | 0.277631 | 0.991491 | 0.492193 | 0.395810 |
| 475.0 | 0.987 | 0.630 | 0.277312 | 0.991468 | 0.491390 | 0.397069 |
| 477.0 | 0.987 | 0.632 | 0.276677 | 0.991422 | 0.489783 | 0.399593 |
| 479.5 | 0.987 | 0.633 | 0.276361 | 0.991399 | 0.488980 | 0.400858 |
| 481.5 | 0.987 | 0.635 | 0.275731 | 0.991352 | 0.487373 | 0.403394 |
| 482.5 | 0.987 | 0.638 | 0.274790 | 0.991281 | 0.484964 | 0.407213 |
| 484.5 | 0.987 | 0.640 | 0.274167 | 0.991233 | 0.483357 | 0.409769 |
| 486.5 | 0.987 | 0.641 | 0.273857 | 0.991209 | 0.482554 | 0.411050 |
| 487.5 | 0.987 | 0.643 | 0.273238 | 0.991160 | 0.480947 | 0.413618 |
| 489.5 | 0.987 | 0.646 | 0.272314 | 0.991086 | 0.478538 | 0.417485 |
| 492.0 | 0.987 | 0.647 | 0.272008 | 0.991061 | 0.477734 | 0.418778 |
| 493.5 | 0.987 | 0.649 | 0.271397 | 0.991010 | 0.476128 | 0.421370 |
| 494.5 | 0.987 | 0.652 | 0.270486 | 0.990933 | 0.473718 | 0.425273 |
| 495.5 | 0.987 | 0.655 | 0.269581 | 0.990855 | 0.471308 | 0.429194 |
| 496.5 | 0.987 | 0.657 | 0.268981 | 0.990802 | 0.469702 | 0.431818 |
| 498.0 | 0.987 | 0.658 | 0.268682 | 0.990776 | 0.468898 | 0.433133 |
| 499.5 | 0.987 | 0.660 | 0.268086 | 0.990722 | 0.467292 | 0.435769 |
| 500.5 | 0.987 | 0.661 | 0.267789 | 0.990695 | 0.466489 | 0.437090 |
| 501.5 | 0.987 | 0.663 | 0.267197 | 0.990640 | 0.464882 | 0.439738 |
| 502.5 | 0.987 | 0.665 | 0.266608 | 0.990585 | 0.463276 | 0.442394 |
| 503.5 | 0.987 | 0.666 | 0.266314 | 0.990557 | 0.462472 | 0.443725 |
| 505.0 | 0.987 | 0.671 | 0.264855 | 0.990415 | 0.458456 | 0.450410 |
| 507.5 | 0.987 | 0.677 | 0.263126 | 0.990238 | 0.453637 | 0.458498 |
| 511.0 | 0.987 | 0.679 | 0.262554 | 0.990178 | 0.452030 | 0.461210 |
| 514.0 | 0.987 | 0.680 | 0.262269 | 0.990148 | 0.451227 | 0.462569 |
| 517.5 | 0.987 | 0.683 | 0.261419 | 0.990055 | 0.448817 | 0.466658 |
| 521.0 | 0.987 | 0.685 | 0.260854 | 0.989993 | 0.447211 | 0.469394 |
| 522.5 | 0.987 | 0.686 | 0.260573 | 0.989961 | 0.446407 | 0.470765 |
| 525.0 | 0.987 | 0.688 | 0.260013 | 0.989898 | 0.444801 | 0.473513 |
| 528.0 | 0.987 | 0.690 | 0.259455 | 0.989833 | 0.443194 | 0.476269 |
| 529.5 | 0.987 | 0.691 | 0.259176 | 0.989801 | 0.442391 | 0.477650 |

Supplementary Table 1 (Continued)

| Cut-point* | Sensitivity | 1 - Specificity | Positive  Predictive  Value | Negative Predictive Value | Accuracy | d^2^ |
| --- | --- | --- | --- | --- | --- | --- |
| 536.5 | 0.987 | 0.694 | 0.258346 | 0.989702 | 0.439981 | 0.481805 |
| 543.0 | 0.987 | 0.696 | 0.257795 | 0.989635 | 0.438375 | 0.484585 |
| 546.0 | 0.987 | 0.697 | 0.257520 | 0.989601 | 0.437571 | 0.485978 |
| 548.5 | 0.987 | 0.699 | 0.256972 | 0.989532 | 0.435965 | 0.488770 |
| 553.5 | 0.987 | 0.700 | 0.256700 | 0.989498 | 0.435162 | 0.490169 |
| 559.0 | 0.987 | 0.702 | 0.256156 | 0.989428 | 0.433555 | 0.492973 |
| 561.5 | 0.987 | 0.704 | 0.255614 | 0.989357 | 0.431949 | 0.495785 |
| 562.5 | 0.987 | 0.708 | 0.254537 | 0.989213 | 0.428736 | 0.501433 |
| 566.0 | 0.987 | 0.710 | 0.254002 | 0.989140 | 0.427129 | 0.504269 |
| 570.5 | 0.987 | 0.711 | 0.253736 | 0.989102 | 0.426326 | 0.505690 |
| 572.5 | 0.987 | 0.713 | 0.253204 | 0.989027 | 0.424719 | 0.508538 |
| 574.0 | 0.987 | 0.715 | 0.252675 | 0.988951 | 0.423113 | 0.511394 |
| 577.5 | 0.987 | 0.718 | 0.251885 | 0.988835 | 0.420703 | 0.515693 |
| 581.0 | 0.987 | 0.721 | 0.251100 | 0.988716 | 0.418293 | 0.520010 |
| 584.5 | 0.987 | 0.722 | 0.250840 | 0.988676 | 0.417490 | 0.521453 |
| 588.0 | 0.987 | 0.724 | 0.250320 | 0.988595 | 0.415883 | 0.524345 |
| 589.5 | 0.987 | 0.725 | 0.250061 | 0.988554 | 0.415080 | 0.525794 |
| 590.5 | 0.987 | 0.727 | 0.249545 | 0.988471 | 0.413474 | 0.528698 |
| 592.0 | 0.987 | 0.729 | 0.249031 | 0.988387 | 0.411867 | 0.531610 |
| 595.0 | 0.987 | 0.730 | 0.248775 | 0.988345 | 0.411064 | 0.533069 |
| 599.5 | 0.987 | 0.732 | 0.248264 | 0.988259 | 0.409457 | 0.535993 |
| 603.0 | 0.987 | 0.733 | 0.248009 | 0.988215 | 0.408654 | 0.537458 |
| 605.0 | 0.987 | 0.736 | 0.247248 | 0.988083 | 0.406244 | 0.541865 |
| 606.5 | 0.987 | 0.738 | 0.246743 | 0.987993 | 0.404638 | 0.544813 |
| 607.5 | 0.987 | 0.741 | 0.245990 | 0.987856 | 0.402228 | 0.549250 |
| 609.0 | 0.987 | 0.743 | 0.245490 | 0.987762 | 0.400622 | 0.552218 |
| 613.0 | 0.987 | 0.744 | 0.245241 | 0.987715 | 0.399818 | 0.553705 |
| 616.5 | 0.987 | 0.746 | 0.244745 | 0.987619 | 0.398212 | 0.556685 |
| 617.5 | 0.987 | 0.747 | 0.244497 | 0.987571 | 0.397409 | 0.558178 |
| 621.5 | 0.987 | 0.749 | 0.244004 | 0.987473 | 0.395802 | 0.561170 |
| 625.5 | 0.987 | 0.750 | 0.243758 | 0.987424 | 0.394999 | 0.562669 |
| 626.5 | 0.987 | 0.752 | 0.243267 | 0.987324 | 0.393392 | 0.565673 |
| 627.5 | 0.987 | 0.754 | 0.242779 | 0.987222 | 0.391786 | 0.568685 |
| 630.0 | 0.987 | 0.757 | 0.242049 | 0.987066 | 0.389376 | 0.573218 |
| 632.5 | 0.987 | 0.758 | 0.241807 | 0.987013 | 0.388573 | 0.574733 |
| 633.5 | 0.987 | 0.760 | 0.241324 | 0.986907 | 0.386966 | 0.577769 |
| 635.0 | 0.987 | 0.761 | 0.241084 | 0.986853 | 0.386163 | 0.579290 |
| 637.5 | 0.987 | 0.763 | 0.240604 | 0.986743 | 0.384556 | 0.582338 |
| 639.5 | 0.987 | 0.766 | 0.239888 | 0.986575 | 0.382147 | 0.586925 |
| 641.5 | 0.987 | 0.768 | 0.239412 | 0.986461 | 0.380540 | 0.589993 |
| 644.0 | 0.987 | 0.774 | 0.237998 | 0.986107 | 0.375721 | 0.599245 |
| 645.5 | 0.987 | 0.775 | 0.237764 | 0.986046 | 0.374917 | 0.600794 |
| 647.0 | 0.987 | 0.777 | 0.237297 | 0.985923 | 0.373311 | 0.603898 |
| 648.5 | 0.987 | 0.780 | 0.236601 | 0.985733 | 0.370901 | 0.608569 |
| 649.5 | 0.987 | 0.783 | 0.235908 | 0.985539 | 0.368491 | 0.613258 |
| 651.0 | 0.987 | 0.785 | 0.235448 | 0.985406 | 0.366885 | 0.616394 |

Supplementary Table 1 (Continued)

| Cut-point* | Sensitivity | 1 - Specificity | Positive  Predictive  Value | Negative Predictive Value | Accuracy | d^2^ |
| --- | --- | --- | --- | --- | --- | --- |
| 654.0 | 0.987 | 0.788 | 0.234763 | 0.985203 | 0.364475 | 0.621113 |
| 656.5 | 0.987 | 0.789 | 0.234535 | 0.985134 | 0.363672 | 0.622690 |
| 659.5 | 0.987 | 0.791 | 0.234081 | 0.984994 | 0.362065 | 0.625850 |
| 662.0 | 0.987 | 0.793 | 0.233628 | 0.984851 | 0.360459 | 0.629018 |
| 663.5 | 0.987 | 0.794 | 0.233403 | 0.984779 | 0.359655 | 0.630605 |
| 664.5 | 0.987 | 0.796 | 0.232953 | 0.984632 | 0.358049 | 0.633785 |
| 665.5 | 0.987 | 0.797 | 0.232728 | 0.984557 | 0.357246 | 0.635378 |
| 667.0 | 0.987 | 0.799 | 0.232281 | 0.984406 | 0.355639 | 0.638570 |
| 670.0 | 0.987 | 0.800 | 0.232058 | 0.984329 | 0.354836 | 0.640169 |
| 672.5 | 0.987 | 0.802 | 0.231614 | 0.984173 | 0.353229 | 0.643373 |
| 675.0 | 0.987 | 0.803 | 0.231392 | 0.984094 | 0.352426 | 0.644978 |
| 678.0 | 0.987 | 0.805 | 0.230950 | 0.983934 | 0.350820 | 0.648194 |
| 679.5 | 0.987 | 0.810 | 0.229852 | 0.983518 | 0.346803 | 0.656269 |
| 683.5 | 0.987 | 0.814 | 0.228981 | 0.983169 | 0.343590 | 0.662765 |
| 687.5 | 0.987 | 0.817 | 0.228332 | 0.982898 | 0.341180 | 0.667658 |
| 688.5 | 0.987 | 0.819 | 0.227902 | 0.982712 | 0.339574 | 0.670930 |
| 690.0 | 0.987 | 0.821 | 0.227473 | 0.982523 | 0.337967 | 0.674210 |
| 694.0 | 0.987 | 0.824 | 0.226832 | 0.982230 | 0.335558 | 0.679145 |
| 697.5 | 0.987 | 0.825 | 0.226620 | 0.982130 | 0.334754 | 0.680794 |
| 699.0 | 0.987 | 0.827 | 0.226196 | 0.981927 | 0.333148 | 0.684098 |
| 700.5 | 0.987 | 0.828 | 0.225984 | 0.981824 | 0.332345 | 0.685753 |
| 701.5 | 0.987 | 0.832 | 0.225142 | 0.981400 | 0.329132 | 0.692393 |
| 702.5 | 0.987 | 0.833 | 0.224933 | 0.981290 | 0.328328 | 0.694058 |
| 704.5 | 0.987 | 0.835 | 0.224515 | 0.981068 | 0.326722 | 0.697394 |
| 706.5 | 0.987 | 0.836 | 0.224307 | 0.980955 | 0.325919 | 0.699065 |
| 707.5 | 0.987 | 0.838 | 0.223891 | 0.980724 | 0.324312 | 0.702413 |
| 710.5 | 0.987 | 0.839 | 0.223684 | 0.980607 | 0.323509 | 0.704090 |
| 717.0 | 0.994 | 0.839 | 0.224914 | 0.990955 | 0.324886 | 0.703957 |
| 723.0 | 0.994 | 0.841 | 0.224499 | 0.990842 | 0.323279 | 0.707317 |
| 725.5 | 0.994 | 0.842 | 0.224292 | 0.990785 | 0.322476 | 0.709000 |
| 729.5 | 0.994 | 0.844 | 0.223880 | 0.990668 | 0.320870 | 0.712372 |
| 733.5 | 0.994 | 0.846 | 0.223469 | 0.990547 | 0.319263 | 0.715752 |
| 735.5 | 0.994 | 0.847 | 0.223264 | 0.990486 | 0.318460 | 0.717445 |
| 738.0 | 0.994 | 0.849 | 0.222855 | 0.990361 | 0.316853 | 0.720837 |
| 740.5 | 0.994 | 0.850 | 0.222651 | 0.990298 | 0.316050 | 0.722536 |
| 747.0 | 0.994 | 0.852 | 0.222245 | 0.990168 | 0.314444 | 0.725940 |
| 754.0 | 0.994 | 0.853 | 0.222042 | 0.990102 | 0.313640 | 0.727645 |
| 757.0 | 0.994 | 0.855 | 0.221638 | 0.989967 | 0.312034 | 0.731061 |
| 761.5 | 0.994 | 0.858 | 0.221034 | 0.989757 | 0.309624 | 0.736200 |
| 765.5 | 0.994 | 0.860 | 0.220633 | 0.989612 | 0.308018 | 0.739636 |
| 766.5 | 0.994 | 0.861 | 0.220434 | 0.989538 | 0.307214 | 0.741357 |
| 768.0 | 0.994 | 0.863 | 0.220035 | 0.989387 | 0.305608 | 0.744805 |
| 769.5 | 0.994 | 0.864 | 0.219837 | 0.989310 | 0.304805 | 0.746532 |
| 773.5 | 0.994 | 0.866 | 0.219440 | 0.989152 | 0.303198 | 0.749992 |
| 778.0 | 0.994 | 0.867 | 0.219243 | 0.989071 | 0.302395 | 0.751725 |
| 781.0 | 0.994 | 0.869 | 0.218848 | 0.988906 | 0.300788 | 0.755197 |

Supplementary Table 1 (Continued)

| Cut-point* | Sensitivity | 1 - Specificity | Positive  Predictive  Value | Negative Predictive Value | Accuracy | d^2^ |
| --- | --- | --- | --- | --- | --- | --- |
| 786.0 | 0.994 | 0.872 | 0.218260 | 0.988649 | 0.298378 | 0.760420 |
| 788.0 | 0.994 | 0.874 | 0.217869 | 0.988471 | 0.296772 | 0.763912 |
| 789.5 | 0.994 | 0.875 | 0.217674 | 0.988380 | 0.295969 | 0.765661 |
| 792.5 | 0.994 | 0.877 | 0.217286 | 0.988193 | 0.294362 | 0.769165 |
| 796.5 | 0.994 | 0.878 | 0.217092 | 0.988098 | 0.293559 | 0.770920 |
| 799.0 | 0.994 | 0.880 | 0.216706 | 0.987902 | 0.291952 | 0.774436 |
| 801.0 | 0.994 | 0.883 | 0.216129 | 0.987595 | 0.289543 | 0.779725 |
| 802.5 | 0.994 | 0.885 | 0.215746 | 0.987382 | 0.287936 | 0.783261 |
| 805.0 | 0.994 | 0.888 | 0.215173 | 0.987049 | 0.285526 | 0.788580 |
| 808.5 | 0.994 | 0.889 | 0.214983 | 0.986934 | 0.284723 | 0.790357 |
| 810.5 | 0.994 | 0.891 | 0.214604 | 0.986697 | 0.283117 | 0.793917 |
| 812.0 | 0.994 | 0.892 | 0.214415 | 0.986575 | 0.282313 | 0.795700 |
| 813.5 | 0.994 | 0.894 | 0.214038 | 0.986326 | 0.280707 | 0.799272 |
| 816.0 | 0.994 | 0.895 | 0.213850 | 0.986197 | 0.279904 | 0.801061 |
| 819.5 | 0.994 | 0.897 | 0.213475 | 0.985933 | 0.278297 | 0.804645 |
| 821.5 | 0.994 | 0.899 | 0.213102 | 0.985658 | 0.276690 | 0.808237 |
| 824.0 | 0.994 | 0.900 | 0.212915 | 0.985517 | 0.275887 | 0.810036 |
| 829.0 | 0.994 | 0.902 | 0.212544 | 0.985226 | 0.274281 | 0.813640 |
| 833.0 | 0.994 | 0.903 | 0.212358 | 0.985076 | 0.273477 | 0.815445 |
| 835.0 | 0.994 | 0.905 | 0.211988 | 0.984766 | 0.271871 | 0.819061 |
| 836.5 | 0.994 | 0.906 | 0.211804 | 0.984607 | 0.271068 | 0.820872 |
| 838.0 | 0.994 | 0.908 | 0.211436 | 0.984277 | 0.269461 | 0.824500 |
| 840.0 | 0.994 | 0.910 | 0.211069 | 0.983934 | 0.267855 | 0.828136 |
| 842.0 | 0.994 | 0.911 | 0.210887 | 0.983756 | 0.267051 | 0.829957 |
| 847.0 | 0.994 | 0.913 | 0.210522 | 0.983389 | 0.265445 | 0.833605 |
| 852.0 | 0.994 | 0.916 | 0.209977 | 0.982806 | 0.263035 | 0.839092 |
| 857.0 | 0.994 | 0.917 | 0.209796 | 0.982602 | 0.262232 | 0.840925 |
| 861.5 | 0.994 | 0.919 | 0.209435 | 0.982180 | 0.260625 | 0.844597 |
| 863.0 | 0.994 | 0.920 | 0.209255 | 0.981962 | 0.259822 | 0.846436 |
| 866.0 | 0.994 | 0.924 | 0.208538 | 0.981030 | 0.256609 | 0.853812 |
| 872.5 | 0.994 | 0.925 | 0.208360 | 0.980782 | 0.255806 | 0.855661 |
| 878.0 | 0.994 | 0.927 | 0.208004 | 0.980266 | 0.254199 | 0.859365 |
| 884.0 | 0.994 | 0.928 | 0.207826 | 0.979997 | 0.253396 | 0.861220 |
| 892.5 | 0.994 | 0.930 | 0.207472 | 0.979438 | 0.251789 | 0.864936 |
| 896.5 | 0.994 | 0.931 | 0.207295 | 0.979146 | 0.250986 | 0.866797 |
| 897.5 | 0.994 | 0.934 | 0.206767 | 0.978219 | 0.248576 | 0.872392 |
| 902.5 | 0.994 | 0.936 | 0.206417 | 0.977553 | 0.246970 | 0.876132 |
| 908.0 | 0.994 | 0.938 | 0.206067 | 0.976846 | 0.245363 | 0.879880 |
| 912.0 | 0.994 | 0.939 | 0.205893 | 0.976475 | 0.244560 | 0.881757 |
| 915.5 | 0.994 | 0.941 | 0.205545 | 0.975697 | 0.242954 | 0.885517 |
| 916.5 | 0.994 | 0.942 | 0.205372 | 0.975289 | 0.242150 | 0.887400 |
| 921.0 | 0.994 | 0.945 | 0.204853 | 0.973976 | 0.239741 | 0.893061 |
| 929.0 | 0.994 | 0.947 | 0.204509 | 0.973020 | 0.238134 | 0.896845 |
| 934.5 | 0.994 | 0.949 | 0.204166 | 0.971992 | 0.236528 | 0.900637 |
| 937.0 | 0.994 | 0.950 | 0.203995 | 0.971448 | 0.235724 | 0.902536 |
| 938.5 | 0.994 | 0.952 | 0.203654 | 0.970293 | 0.234118 | 0.906340 |

Supplementary Table 1 (Continued)

| Cut-point* | Sensitivity | 1 - Specificity | Positive  Predictive  Value | Negative Predictive Value | Accuracy | d^2^ |
| --- | --- | --- | --- | --- | --- | --- |
| 961.5 | 0.994 | 0.956 | 0.202975 | 0.967680 | 0.230905 | 0.913972 |
| 965.0 | 0.994 | 0.958 | 0.202637 | 0.966193 | 0.229298 | 0.917800 |
| 977.5 | 0.994 | 0.959 | 0.202468 | 0.965397 | 0.228495 | 0.919717 |
| 988.5 | 0.994 | 0.961 | 0.202132 | 0.963687 | 0.226888 | 0.923557 |
| 995.5 | 0.994 | 0.963 | 0.201797 | 0.961799 | 0.225282 | 0.927405 |
| 1003.0 | 0.994 | 0.964 | 0.201630 | 0.960779 | 0.224479 | 0.929332 |
| 1008.0 | 0.994 | 0.966 | 0.201297 | 0.958568 | 0.222872 | 0.933192 |
| 1017.0 | 0.994 | 0.967 | 0.201130 | 0.957366 | 0.222069 | 0.935125 |
| 1028.5 | 0.994 | 0.969 | 0.200799 | 0.954740 | 0.220462 | 0.938997 |
| 1037.0 | 0.994 | 0.970 | 0.200633 | 0.953302 | 0.219659 | 0.940936 |
| 1047.0 | 0.994 | 0.972 | 0.200303 | 0.950132 | 0.218053 | 0.944820 |
| 1056.5 | 0.994 | 0.973 | 0.200138 | 0.948381 | 0.217249 | 0.946765 |
| 1077.0 | 0.994 | 0.975 | 0.199810 | 0.944480 | 0.215643 | 0.950661 |
| 1097.5 | 0.994 | 0.977 | 0.199482 | 0.939943 | 0.214036 | 0.954565 |
| 1113.0 | 0.994 | 0.978 | 0.199319 | 0.937384 | 0.213233 | 0.956520 |
| 1132.5 | 0.994 | 0.980 | 0.198993 | 0.931551 | 0.211627 | 0.960436 |
| 1166.5 | 0.994 | 0.981 | 0.198831 | 0.928207 | 0.210823 | 0.962397 |
| 1196.5 | 0.994 | 0.983 | 0.198506 | 0.920432 | 0.209217 | 0.966325 |
| 1210.0 | 0.994 | 0.984 | 0.198345 | 0.915878 | 0.208414 | 0.968292 |
| 1245.5 | 0.994 | 0.986 | 0.198022 | 0.905002 | 0.206807 | 0.972232 |
| 1273.5 | 0.994 | 0.988 | 0.197700 | 0.890896 | 0.205201 | 0.976180 |
| 1371.5 | 0.994 | 0.989 | 0.197540 | 0.882147 | 0.204397 | 0.978157 |
| 1500.5 | 0.994 | 0.991 | 0.197220 | 0.859633 | 0.202791 | 0.982117 |
| 1587.0 | 0.994 | 0.992 | 0.197060 | 0.844811 | 0.201987 | 0.984100 |
| 1740.0 | 0.994 | 0.994 | 0.196742 | 0.803258 | 0.200381 | 0.988072 |
| 2028.0 | 1.000 | 0.994 | 0.197695 | 1.000000 | 0.201561 | 0.988036 |
| 2247.5 | 1.000 | 0.995 | 0.197535 | 1.000000 | 0.200758 | 0.990025 |
| 2342.5 | 1.000 | 0.997 | 0.197217 | 1.000000 | 0.199152 | 0.994009 |
| 2530.0 | 1.000 | 0.998 | 0.197058 | 1.000000 | 0.198348 | 0.996004 |
| 2657.0 | 1.000 | 1.000 | 0.196742 | 0.000000 | 0.196742 | 1.000000 |
