# Supplementary material for: Measuring physical activity in older adults: calibrating cut-points for the MotionWatch 8©
Source: Front Aging Neurosci. 2015 Aug 25;7:165. doi: 10.3389/fnagi.2015.00165 (PMC4548198; doi:10.3389/fnagi.2015.00165)
Supplement: Supplementary file 2 [file Table_2.DOCX]

Supplementary Table 2: Characteristics of Each Potential Cut-point for Moderate-to-Vigorous Physical Activity

**Note: Cut-points for moderate-to-vigorous physical activity is for all counts greater than or equal to the given value*

| Cut-point* | Sensitivity | 1 – Specificity | Positive Predictive Value | Negative Predictive Value | Accuracy | d^2^ |
| --- | --- | --- | --- | --- | --- | --- |
| 1.0 | 1.000 | 1.000 | 0.45614 | 0.00000 | 0.456140 | 1.000000 |
| 2.0 | 0.992 | 0.880 | 0.485981 | 0.94705 | 0.517754 | 0.774464 |
| 4.5 | 0.992 | 0.864 | 0.490566 | 0.95298 | 0.526456 | 0.746560 |
| 5.5 | 0.992 | 0.857 | 0.492599 | 0.95518 | 0.530263 | 0.734513 |
| 6.5 | 0.992 | 0.855 | 0.493183 | 0.95577 | 0.531351 | 0.731089 |
| 7.5 | 0.992 | 0.853 | 0.493769 | 0.95635 | 0.532439 | 0.727673 |
| 8.5 | 0.986 | 0.853 | 0.492252 | 0.92603 | 0.529702 | 0.727805 |
| 9.5 | 0.986 | 0.848 | 0.493722 | 0.92829 | 0.532421 | 0.719300 |
| 10.5 | 0.986 | 0.843 | 0.495200 | 0.93041 | 0.535140 | 0.710845 |
| 12.0 | 0.986 | 0.834 | 0.497883 | 0.93394 | 0.540035 | 0.695752 |
| 13.5 | 0.984 | 0.825 | 0.500088 | 0.92878 | 0.544018 | 0.680881 |
| 14.5 | 0.981 | 0.820 | 0.500844 | 0.91867 | 0.545368 | 0.672761 |
| 15.5 | 0.978 | 0.820 | 0.500079 | 0.90702 | 0.544000 | 0.672884 |
| 16.5 | 0.978 | 0.809 | 0.503455 | 0.91191 | 0.549982 | 0.654965 |
| 17.5 | 0.978 | 0.802 | 0.505627 | 0.91475 | 0.553789 | 0.643688 |
| 18.5 | 0.973 | 0.800 | 0.504970 | 0.89829 | 0.552596 | 0.640729 |
| 19.5 | 0.973 | 0.793 | 0.507167 | 0.90139 | 0.556404 | 0.629578 |
| 21.0 | 0.973 | 0.786 | 0.509383 | 0.90431 | 0.560211 | 0.618525 |
| 22.5 | 0.973 | 0.779 | 0.511619 | 0.90706 | 0.564018 | 0.607570 |
| 23.5 | 0.967 | 0.774 | 0.511682 | 0.89090 | 0.564000 | 0.600165 |
| 24.5 | 0.967 | 0.770 | 0.512976 | 0.89259 | 0.566175 | 0.593989 |
| 25.5 | 0.964 | 0.770 | 0.512200 | 0.88396 | 0.564807 | 0.594196 |
| 26.5 | 0.959 | 0.770 | 0.510901 | 0.86994 | 0.562526 | 0.594581 |
| 27.5 | 0.956 | 0.767 | 0.511093 | 0.86327 | 0.562789 | 0.590225 |
| 29.0 | 0.951 | 0.763 | 0.511090 | 0.85222 | 0.562684 | 0.584570 |
| 30.5 | 0.951 | 0.758 | 0.512732 | 0.85483 | 0.565404 | 0.576965 |
| 31.5 | 0.951 | 0.753 | 0.514386 | 0.85735 | 0.568123 | 0.569410 |
| 32.5 | 0.951 | 0.749 | 0.515716 | 0.85930 | 0.570298 | 0.563402 |
| 33.5 | 0.951 | 0.744 | 0.517389 | 0.86167 | 0.573018 | 0.555937 |
| 34.5 | 0.951 | 0.735 | 0.520427 | 0.86574 | 0.577912 | 0.542626 |
| 35.5 | 0.948 | 0.735 | 0.519638 | 0.85868 | 0.576544 | 0.542929 |
| 36.5 | 0.942 | 0.728 | 0.520442 | 0.84829 | 0.577614 | 0.533348 |
| 37.5 | 0.942 | 0.724 | 0.521817 | 0.85016 | 0.579789 | 0.527540 |
| 38.5 | 0.942 | 0.719 | 0.523546 | 0.85243 | 0.582509 | 0.520325 |
| 39.5 | 0.942 | 0.712 | 0.525986 | 0.85550 | 0.586316 | 0.510308 |
| 41.0 | 0.940 | 0.712 | 0.525456 | 0.85126 | 0.585404 | 0.510544 |
| 42.5 | 0.940 | 0.707 | 0.527213 | 0.85342 | 0.588123 | 0.503449 |
| 44.0 | 0.940 | 0.703 | 0.528627 | 0.85511 | 0.590298 | 0.497809 |
| 45.5 | 0.940 | 0.700 | 0.529692 | 0.85635 | 0.591930 | 0.493600 |
| 46.5 | 0.934 | 0.700 | 0.528097 | 0.84423 | 0.589193 | 0.494356 |
| 47.5 | 0.934 | 0.698 | 0.528810 | 0.84510 | 0.590281 | 0.491560 |

Supplementary Table 2 (Continued)

| Cut-point* | Sensitivity | 1 – Specificity | Positive Predictive Value | Negative Predictive Value | Accuracy | d^2^ |
| --- | --- | --- | --- | --- | --- | --- |
| 49.5 | 0.934 | 0.689 | 0.532042 | 0.84890 | 0.595175 | 0.479077 |
| 50.5 | 0.934 | 0.684 | 0.533855 | 0.85094 | 0.597895 | 0.472212 |
| 51.5 | 0.931 | 0.684 | 0.533054 | 0.84521 | 0.596526 | 0.472617 |
| 52.5 | 0.931 | 0.682 | 0.533783 | 0.84604 | 0.597614 | 0.469885 |
| 54.0 | 0.929 | 0.680 | 0.533979 | 0.84311 | 0.597789 | 0.467441 |
| 55.5 | 0.929 | 0.677 | 0.535079 | 0.84434 | 0.599421 | 0.463370 |
| 56.5 | 0.929 | 0.675 | 0.535815 | 0.84515 | 0.600509 | 0.460666 |
| 57.5 | 0.929 | 0.671 | 0.537293 | 0.84674 | 0.602684 | 0.455282 |
| 58.5 | 0.926 | 0.671 | 0.536489 | 0.84129 | 0.601316 | 0.455717 |
| 59.5 | 0.926 | 0.668 | 0.537603 | 0.84250 | 0.602947 | 0.451700 |
| 60.5 | 0.926 | 0.666 | 0.538348 | 0.84330 | 0.604035 | 0.449032 |
| 61.5 | 0.926 | 0.661 | 0.540220 | 0.84525 | 0.606754 | 0.442397 |
| 62.5 | 0.923 | 0.657 | 0.540922 | 0.84155 | 0.607561 | 0.437578 |
| 63.5 | 0.923 | 0.650 | 0.543581 | 0.84423 | 0.611368 | 0.428429 |
| 64.5 | 0.918 | 0.647 | 0.543381 | 0.83694 | 0.610719 | 0.425333 |
| 65.5 | 0.915 | 0.645 | 0.543337 | 0.83277 | 0.610439 | 0.423250 |
| 66.5 | 0.915 | 0.641 | 0.544880 | 0.83432 | 0.612614 | 0.418106 |
| 68.0 | 0.915 | 0.638 | 0.546043 | 0.83547 | 0.614246 | 0.414269 |
| 70.0 | 0.915 | 0.636 | 0.546821 | 0.83622 | 0.615333 | 0.411721 |
| 71.5 | 0.912 | 0.634 | 0.546788 | 0.83218 | 0.615053 | 0.409700 |
| 72.5 | 0.909 | 0.631 | 0.547147 | 0.82861 | 0.615316 | 0.406442 |
| 73.5 | 0.909 | 0.627 | 0.548722 | 0.83014 | 0.617491 | 0.401410 |
| 74.5 | 0.909 | 0.624 | 0.549909 | 0.83127 | 0.619123 | 0.397657 |
| 75.5 | 0.909 | 0.615 | 0.553502 | 0.83456 | 0.624018 | 0.386506 |
| 76.5 | 0.909 | 0.611 | 0.555115 | 0.83598 | 0.626193 | 0.381602 |
| 77.5 | 0.909 | 0.599 | 0.560008 | 0.84010 | 0.632719 | 0.367082 |
| 79.0 | 0.909 | 0.594 | 0.562072 | 0.84176 | 0.635439 | 0.361117 |
| 80.5 | 0.907 | 0.592 | 0.562360 | 0.83951 | 0.635614 | 0.359113 |
| 81.5 | 0.907 | 0.590 | 0.563193 | 0.84016 | 0.636702 | 0.356749 |
| 83.5 | 0.904 | 0.590 | 0.562377 | 0.83585 | 0.635333 | 0.357316 |
| 86.0 | 0.901 | 0.590 | 0.561559 | 0.83159 | 0.633965 | 0.357901 |
| 87.5 | 0.898 | 0.588 | 0.561574 | 0.82806 | 0.633684 | 0.356148 |
| 89.0 | 0.898 | 0.585 | 0.562833 | 0.82909 | 0.635316 | 0.352629 |
| 90.5 | 0.896 | 0.583 | 0.563127 | 0.82701 | 0.635491 | 0.350705 |
| 91.5 | 0.896 | 0.578 | 0.565245 | 0.82871 | 0.638211 | 0.344900 |
| 92.5 | 0.896 | 0.576 | 0.566096 | 0.82938 | 0.639298 | 0.342592 |
| 94.0 | 0.896 | 0.574 | 0.566951 | 0.83004 | 0.640386 | 0.340292 |
| 95.5 | 0.896 | 0.571 | 0.568237 | 0.83103 | 0.642018 | 0.336857 |
| 97.0 | 0.896 | 0.569 | 0.569097 | 0.83168 | 0.643105 | 0.334577 |
| 99.0 | 0.896 | 0.567 | 0.569961 | 0.83233 | 0.644193 | 0.332305 |
| 101.0 | 0.896 | 0.560 | 0.573003 | 0.83456 | 0.648000 | 0.324416 |
| 102.5 | 0.893 | 0.560 | 0.572182 | 0.83059 | 0.646632 | 0.325049 |
| 103.5 | 0.890 | 0.558 | 0.572234 | 0.82732 | 0.646351 | 0.323464 |
| 104.5 | 0.890 | 0.553 | 0.574436 | 0.82892 | 0.649070 | 0.317909 |
| 107.0 | 0.890 | 0.551 | 0.575321 | 0.82955 | 0.650158 | 0.315701 |
| 109.5 | 0.890 | 0.546 | 0.577547 | 0.83111 | 0.652877 | 0.310216 |

Supplementary Table 2 (Continued)

| Cut-point* | Sensitivity | 1 – Specificity | Positive Predictive Value | Negative Predictive Value | Accuracy | d^2^ |
| --- | --- | --- | --- | --- | --- | --- |
| 112.5 | 0.890 | 0.539 | 0.580692 | 0.83325 | 0.656684 | 0.302621 |
| 113.5 | 0.890 | 0.535 | 0.582505 | 0.83444 | 0.658860 | 0.298325 |
| 115.0 | 0.890 | 0.528 | 0.585704 | 0.83650 | 0.662667 | 0.290884 |
| 116.5 | 0.887 | 0.525 | 0.586267 | 0.83366 | 0.662930 | 0.288394 |
| 117.5 | 0.887 | 0.518 | 0.589519 | 0.83568 | 0.666737 | 0.281093 |
| 118.5 | 0.885 | 0.518 | 0.588973 | 0.83326 | 0.665825 | 0.281549 |
| 119.5 | 0.885 | 0.516 | 0.589909 | 0.83383 | 0.666912 | 0.279481 |
| 121.0 | 0.882 | 0.516 | 0.589088 | 0.83023 | 0.665544 | 0.280180 |
| 123.5 | 0.879 | 0.514 | 0.589203 | 0.82726 | 0.665263 | 0.278837 |
| 125.5 | 0.879 | 0.512 | 0.590146 | 0.82784 | 0.666351 | 0.276785 |
| 126.5 | 0.876 | 0.512 | 0.589319 | 0.82432 | 0.664982 | 0.277520 |
| 127.5 | 0.876 | 0.509 | 0.590741 | 0.82521 | 0.666614 | 0.274457 |
| 128.5 | 0.876 | 0.507 | 0.591692 | 0.82580 | 0.667702 | 0.272425 |
| 129.5 | 0.876 | 0.502 | 0.594084 | 0.82724 | 0.670421 | 0.267380 |
| 131.5 | 0.874 | 0.498 | 0.595461 | 0.82610 | 0.671684 | 0.263880 |
| 134.5 | 0.874 | 0.495 | 0.596916 | 0.82695 | 0.673316 | 0.260901 |
| 136.5 | 0.874 | 0.488 | 0.600338 | 0.82891 | 0.677123 | 0.254020 |
| 137.5 | 0.874 | 0.486 | 0.601323 | 0.82946 | 0.678211 | 0.252072 |
| 139.0 | 0.874 | 0.484 | 0.602311 | 0.83001 | 0.679298 | 0.250132 |
| 140.5 | 0.868 | 0.479 | 0.603148 | 0.82475 | 0.679281 | 0.246865 |
| 142.0 | 0.868 | 0.475 | 0.605154 | 0.82585 | 0.681456 | 0.243049 |
| 143.5 | 0.865 | 0.475 | 0.604326 | 0.82259 | 0.680088 | 0.243850 |
| 144.5 | 0.865 | 0.468 | 0.607871 | 0.82452 | 0.683895 | 0.237249 |
| 145.5 | 0.863 | 0.465 | 0.608851 | 0.82320 | 0.684614 | 0.234994 |
| 146.5 | 0.860 | 0.465 | 0.608022 | 0.82002 | 0.683246 | 0.235825 |
| 147.5 | 0.860 | 0.463 | 0.609049 | 0.82057 | 0.684333 | 0.233969 |
| 149.0 | 0.860 | 0.461 | 0.610079 | 0.82112 | 0.685421 | 0.232121 |
| 150.5 | 0.860 | 0.459 | 0.611113 | 0.82166 | 0.686509 | 0.230281 |
| 151.5 | 0.857 | 0.459 | 0.610282 | 0.81854 | 0.685140 | 0.231130 |
| 152.5 | 0.857 | 0.456 | 0.611840 | 0.81936 | 0.686772 | 0.228385 |
| 153.5 | 0.857 | 0.454 | 0.612884 | 0.81990 | 0.687860 | 0.226565 |
| 154.5 | 0.857 | 0.452 | 0.613931 | 0.82044 | 0.688947 | 0.224753 |
| 155.5 | 0.854 | 0.449 | 0.614678 | 0.81817 | 0.689211 | 0.222917 |
| 157.5 | 0.854 | 0.438 | 0.620535 | 0.82110 | 0.695193 | 0.213160 |
| 159.5 | 0.852 | 0.435 | 0.621601 | 0.81988 | 0.695912 | 0.211129 |
| 160.5 | 0.849 | 0.435 | 0.620771 | 0.81689 | 0.694544 | 0.212026 |
| 161.5 | 0.849 | 0.433 | 0.621855 | 0.81742 | 0.695632 | 0.210290 |
| 163.0 | 0.849 | 0.429 | 0.624035 | 0.81847 | 0.697807 | 0.206842 |
| 164.5 | 0.849 | 0.424 | 0.626782 | 0.81976 | 0.700526 | 0.202577 |
| 165.5 | 0.849 | 0.422 | 0.627887 | 0.82027 | 0.701614 | 0.200885 |
| 166.5 | 0.849 | 0.419 | 0.629553 | 0.82103 | 0.703246 | 0.198362 |
| 167.5 | 0.849 | 0.417 | 0.630668 | 0.82154 | 0.704333 | 0.196690 |
| 168.5 | 0.849 | 0.415 | 0.631787 | 0.82204 | 0.705421 | 0.195026 |
| 170.5 | 0.846 | 0.415 | 0.630963 | 0.81914 | 0.704053 | 0.195941 |
| 172.5 | 0.841 | 0.410 | 0.632404 | 0.81564 | 0.704491 | 0.193381 |
| 174.0 | 0.838 | 0.410 | 0.631573 | 0.81282 | 0.703123 | 0.194344 |

Supplementary Table 2 (Continued)

| Cut-point* | Sensitivity | 1 – Specificity | Positive Predictive Value | Negative Predictive Value | Accuracy | d^2^ |
| --- | --- | --- | --- | --- | --- | --- |
| 178.5 | 0.838 | 0.406 | 0.633851 | 0.81384 | 0.705298 | 0.191080 |
| 179.5 | 0.835 | 0.406 | 0.633018 | 0.81105 | 0.703930 | 0.192061 |
| 180.5 | 0.832 | 0.403 | 0.633905 | 0.80905 | 0.704193 | 0.190633 |
| 182.0 | 0.830 | 0.399 | 0.635659 | 0.80825 | 0.705456 | 0.188101 |
| 183.5 | 0.827 | 0.399 | 0.634820 | 0.80553 | 0.704088 | 0.189130 |
| 184.5 | 0.824 | 0.394 | 0.636899 | 0.80413 | 0.705439 | 0.186212 |
| 185.5 | 0.824 | 0.392 | 0.638075 | 0.80464 | 0.706526 | 0.184640 |
| 188.0 | 0.821 | 0.392 | 0.637232 | 0.80197 | 0.705158 | 0.185705 |
| 191.0 | 0.819 | 0.392 | 0.636668 | 0.80020 | 0.704246 | 0.186425 |
| 192.5 | 0.819 | 0.389 | 0.638443 | 0.80099 | 0.705877 | 0.184082 |
| 193.5 | 0.816 | 0.387 | 0.638786 | 0.79888 | 0.705596 | 0.183625 |
| 195.0 | 0.813 | 0.387 | 0.637936 | 0.79627 | 0.704228 | 0.184738 |
| 196.5 | 0.813 | 0.382 | 0.640934 | 0.79759 | 0.706947 | 0.180893 |
| 197.5 | 0.813 | 0.380 | 0.642141 | 0.79811 | 0.708035 | 0.179369 |
| 198.5 | 0.813 | 0.378 | 0.643353 | 0.79862 | 0.709123 | 0.177853 |
| 200.5 | 0.813 | 0.376 | 0.644569 | 0.79914 | 0.710211 | 0.176345 |
| 202.5 | 0.813 | 0.373 | 0.646402 | 0.79991 | 0.711842 | 0.174098 |
| 204.5 | 0.808 | 0.371 | 0.646221 | 0.79617 | 0.710649 | 0.174505 |
| 206.5 | 0.805 | 0.371 | 0.645370 | 0.79364 | 0.709281 | 0.175666 |
| 207.5 | 0.802 | 0.364 | 0.648867 | 0.79295 | 0.711719 | 0.171700 |
| 208.5 | 0.799 | 0.364 | 0.648013 | 0.79047 | 0.710351 | 0.172897 |
| 212.0 | 0.799 | 0.362 | 0.649269 | 0.79099 | 0.711439 | 0.171445 |
| 216.0 | 0.797 | 0.362 | 0.648698 | 0.78935 | 0.710526 | 0.172253 |
| 218.0 | 0.797 | 0.357 | 0.651861 | 0.79065 | 0.713246 | 0.168658 |
| 219.5 | 0.794 | 0.355 | 0.652280 | 0.78873 | 0.712965 | 0.168461 |
| 220.5 | 0.791 | 0.350 | 0.654635 | 0.78760 | 0.714316 | 0.166181 |
| 221.5 | 0.791 | 0.348 | 0.655929 | 0.78812 | 0.715404 | 0.164785 |
| 222.5 | 0.791 | 0.343 | 0.659188 | 0.78939 | 0.718123 | 0.161330 |
| 223.5 | 0.788 | 0.343 | 0.658334 | 0.78701 | 0.716754 | 0.162593 |
| 224.5 | 0.788 | 0.341 | 0.659648 | 0.78752 | 0.717842 | 0.161225 |
| 225.5 | 0.788 | 0.336 | 0.662956 | 0.78878 | 0.720561 | 0.157840 |
| 227.0 | 0.788 | 0.334 | 0.664289 | 0.78928 | 0.721649 | 0.156500 |
| 229.0 | 0.786 | 0.334 | 0.663722 | 0.78771 | 0.720737 | 0.157352 |
| 231.0 | 0.783 | 0.334 | 0.662868 | 0.78538 | 0.719368 | 0.158645 |
| 232.5 | 0.780 | 0.329 | 0.665376 | 0.78432 | 0.720719 | 0.156641 |
| 233.5 | 0.777 | 0.329 | 0.664518 | 0.78202 | 0.719351 | 0.157970 |
| 234.5 | 0.777 | 0.327 | 0.665876 | 0.78253 | 0.720439 | 0.156658 |
| 235.5 | 0.775 | 0.327 | 0.665302 | 0.78101 | 0.719526 | 0.157554 |
| 238.0 | 0.769 | 0.327 | 0.663569 | 0.77647 | 0.716789 | 0.160290 |
| 241.0 | 0.769 | 0.320 | 0.668383 | 0.77826 | 0.720596 | 0.155761 |
| 244.5 | 0.769 | 0.318 | 0.669771 | 0.77877 | 0.721684 | 0.154485 |
| 247.5 | 0.769 | 0.316 | 0.671165 | 0.77927 | 0.722772 | 0.153217 |
| 250.5 | 0.769 | 0.311 | 0.674675 | 0.78052 | 0.725491 | 0.150082 |
| 253.5 | 0.769 | 0.309 | 0.676090 | 0.78102 | 0.726579 | 0.148842 |
| 255.0 | 0.766 | 0.309 | 0.675233 | 0.77880 | 0.725211 | 0.150237 |
| 257.0 | 0.761 | 0.306 | 0.675936 | 0.77589 | 0.724561 | 0.150757 |

Supplementary Table 2 (Continued)

| Cut-point* | Sensitivity | 1 – Specificity | Positive Predictive Value | Negative Predictive Value | Accuracy | d^2^ |
| --- | --- | --- | --- | --- | --- | --- |
| 259.0 | 0.761 | 0.302 | 0.678812 | 0.77689 | 0.726737 | 0.148325 |
| 261.0 | 0.758 | 0.302 | 0.677950 | 0.77472 | 0.725368 | 0.149768 |
| 263.0 | 0.755 | 0.300 | 0.678534 | 0.77307 | 0.725088 | 0.150025 |
| 264.5 | 0.753 | 0.300 | 0.677956 | 0.77164 | 0.724175 | 0.151009 |
| 266.5 | 0.753 | 0.297 | 0.680146 | 0.77239 | 0.725807 | 0.149218 |
| 268.5 | 0.750 | 0.297 | 0.679277 | 0.77026 | 0.724439 | 0.150709 |
| 269.5 | 0.750 | 0.295 | 0.680747 | 0.77076 | 0.725526 | 0.149525 |
| 270.5 | 0.747 | 0.288 | 0.685079 | 0.77040 | 0.727965 | 0.146953 |
| 272.5 | 0.747 | 0.281 | 0.690364 | 0.77213 | 0.731772 | 0.142970 |
| 275.0 | 0.745 | 0.281 | 0.689790 | 0.77074 | 0.730860 | 0.143986 |
| 276.5 | 0.745 | 0.279 | 0.691317 | 0.77123 | 0.731947 | 0.142866 |
| 278.0 | 0.745 | 0.276 | 0.693619 | 0.77196 | 0.733579 | 0.141201 |
| 279.5 | 0.745 | 0.274 | 0.695162 | 0.77245 | 0.734667 | 0.140101 |
| 280.5 | 0.742 | 0.274 | 0.694306 | 0.77038 | 0.733298 | 0.141640 |
| 281.5 | 0.742 | 0.272 | 0.695859 | 0.77087 | 0.734386 | 0.140548 |
| 283.0 | 0.736 | 0.272 | 0.694138 | 0.76678 | 0.731649 | 0.143680 |
| 285.0 | 0.736 | 0.270 | 0.695703 | 0.76727 | 0.732737 | 0.142596 |
| 286.5 | 0.734 | 0.270 | 0.695126 | 0.76592 | 0.731825 | 0.143656 |
| 288.0 | 0.728 | 0.270 | 0.693384 | 0.76190 | 0.729088 | 0.146884 |
| 289.5 | 0.725 | 0.267 | 0.694880 | 0.76065 | 0.729351 | 0.146914 |
| 290.5 | 0.723 | 0.265 | 0.695887 | 0.75983 | 0.729526 | 0.146954 |
| 291.5 | 0.723 | 0.260 | 0.699903 | 0.76106 | 0.732246 | 0.144329 |
| 292.5 | 0.723 | 0.258 | 0.701523 | 0.76155 | 0.733333 | 0.143293 |
| 296.0 | 0.720 | 0.256 | 0.702281 | 0.76008 | 0.733053 | 0.143936 |
| 299.5 | 0.720 | 0.253 | 0.704740 | 0.76082 | 0.734684 | 0.142409 |
| 301.5 | 0.717 | 0.247 | 0.708848 | 0.76033 | 0.736579 | 0.141098 |
| 303.5 | 0.714 | 0.247 | 0.707982 | 0.75841 | 0.735211 | 0.142805 |
| 304.5 | 0.712 | 0.247 | 0.707402 | 0.75713 | 0.734298 | 0.143953 |
| 305.5 | 0.709 | 0.247 | 0.706527 | 0.75522 | 0.732930 | 0.145690 |
| 307.0 | 0.706 | 0.244 | 0.708179 | 0.75405 | 0.733193 | 0.145972 |
| 308.5 | 0.706 | 0.242 | 0.709877 | 0.75454 | 0.734281 | 0.145000 |
| 309.5 | 0.706 | 0.240 | 0.711583 | 0.75503 | 0.735368 | 0.144036 |
| 311.5 | 0.706 | 0.237 | 0.714158 | 0.75576 | 0.737000 | 0.142605 |
| 314.5 | 0.703 | 0.237 | 0.713288 | 0.75388 | 0.735632 | 0.144378 |
| 317.0 | 0.701 | 0.237 | 0.712705 | 0.75263 | 0.734719 | 0.145570 |
| 318.5 | 0.698 | 0.235 | 0.713561 | 0.75126 | 0.734439 | 0.146429 |
| 320.5 | 0.695 | 0.235 | 0.712680 | 0.74941 | 0.733070 | 0.148250 |
| 322.5 | 0.690 | 0.235 | 0.711199 | 0.74634 | 0.730789 | 0.151325 |
| 326.0 | 0.687 | 0.235 | 0.710303 | 0.74451 | 0.729421 | 0.153194 |
| 329.5 | 0.687 | 0.233 | 0.712059 | 0.74501 | 0.730509 | 0.152258 |
| 331.0 | 0.684 | 0.230 | 0.713816 | 0.74394 | 0.730772 | 0.152756 |
| 332.5 | 0.684 | 0.224 | 0.719185 | 0.74541 | 0.734035 | 0.150032 |
| 333.5 | 0.681 | 0.224 | 0.718296 | 0.74362 | 0.732667 | 0.151937 |
| 334.5 | 0.679 | 0.221 | 0.720424 | 0.74316 | 0.733386 | 0.151882 |
| 337.5 | 0.673 | 0.221 | 0.718633 | 0.73961 | 0.730649 | 0.155770 |
| 340.5 | 0.670 | 0.219 | 0.719567 | 0.73834 | 0.730368 | 0.156861 |

Supplementary Table 2 (Continued)

| Cut-point* | Sensitivity | 1 – Specificity | Positive Predictive Value | Negative Predictive Value | Accuracy | d^2^ |
| --- | --- | --- | --- | --- | --- | --- |
| 346.5 | 0.665 | 0.217 | 0.719907 | 0.73592 | 0.729175 | 0.159314 |
| 350.0 | 0.659 | 0.217 | 0.718076 | 0.73246 | 0.726439 | 0.163370 |
| 354.0 | 0.657 | 0.214 | 0.720273 | 0.73206 | 0.727158 | 0.163445 |
| 355.5 | 0.654 | 0.214 | 0.719350 | 0.73035 | 0.725789 | 0.165512 |
| 357.5 | 0.654 | 0.212 | 0.721242 | 0.73085 | 0.726877 | 0.164660 |
| 359.5 | 0.654 | 0.210 | 0.723144 | 0.73135 | 0.727965 | 0.163816 |
| 362.0 | 0.651 | 0.210 | 0.722222 | 0.72965 | 0.726596 | 0.165901 |
| 365.5 | 0.651 | 0.207 | 0.725100 | 0.73040 | 0.728228 | 0.164650 |
| 367.5 | 0.648 | 0.207 | 0.724178 | 0.72871 | 0.726860 | 0.166753 |
| 368.5 | 0.643 | 0.205 | 0.724570 | 0.72641 | 0.725667 | 0.169474 |
| 369.5 | 0.643 | 0.203 | 0.726522 | 0.72691 | 0.726754 | 0.168658 |
| 371.5 | 0.640 | 0.203 | 0.725592 | 0.72525 | 0.725386 | 0.170809 |
| 373.5 | 0.635 | 0.200 | 0.726993 | 0.72324 | 0.724737 | 0.173225 |
| 374.5 | 0.629 | 0.196 | 0.729113 | 0.72097 | 0.724175 | 0.176057 |
| 375.5 | 0.624 | 0.189 | 0.734683 | 0.72002 | 0.725702 | 0.177097 |
| 379.5 | 0.621 | 0.189 | 0.733742 | 0.71842 | 0.724333 | 0.179362 |
| 385.0 | 0.621 | 0.184 | 0.738947 | 0.71966 | 0.727053 | 0.177497 |
| 389.0 | 0.618 | 0.184 | 0.738012 | 0.71807 | 0.725684 | 0.179780 |
| 393.0 | 0.618 | 0.182 | 0.740120 | 0.71856 | 0.726772 | 0.179048 |
| 395.5 | 0.613 | 0.180 | 0.740682 | 0.71642 | 0.725579 | 0.182169 |
| 397.0 | 0.610 | 0.180 | 0.739739 | 0.71485 | 0.724211 | 0.184500 |
| 399.0 | 0.607 | 0.180 | 0.738789 | 0.71328 | 0.722842 | 0.186849 |
| 401.0 | 0.604 | 0.180 | 0.737831 | 0.71173 | 0.721474 | 0.189216 |
| 404.0 | 0.604 | 0.177 | 0.741069 | 0.71247 | 0.723105 | 0.188145 |
| 408.0 | 0.602 | 0.177 | 0.740432 | 0.71144 | 0.722193 | 0.189733 |
| 411.0 | 0.602 | 0.175 | 0.742610 | 0.71194 | 0.723281 | 0.189029 |
| 413.0 | 0.599 | 0.175 | 0.741654 | 0.71040 | 0.721912 | 0.191426 |
| 414.5 | 0.593 | 0.173 | 0.741928 | 0.70783 | 0.720263 | 0.195578 |
| 416.0 | 0.591 | 0.173 | 0.741280 | 0.70682 | 0.719351 | 0.197210 |
| 417.5 | 0.591 | 0.171 | 0.743504 | 0.70732 | 0.720439 | 0.196522 |
| 420.0 | 0.588 | 0.168 | 0.745902 | 0.70655 | 0.720702 | 0.197968 |
| 422.5 | 0.588 | 0.164 | 0.750442 | 0.70755 | 0.722877 | 0.196640 |
| 424.0 | 0.585 | 0.161 | 0.752933 | 0.70679 | 0.723140 | 0.198146 |
| 426.0 | 0.585 | 0.159 | 0.755251 | 0.70728 | 0.724228 | 0.197506 |
| 427.5 | 0.580 | 0.159 | 0.753661 | 0.70479 | 0.721947 | 0.201681 |
| 431.0 | 0.574 | 0.157 | 0.754080 | 0.70233 | 0.720298 | 0.206125 |
| 435.0 | 0.571 | 0.157 | 0.753107 | 0.70086 | 0.718930 | 0.208690 |
| 436.5 | 0.569 | 0.157 | 0.752454 | 0.69988 | 0.718018 | 0.210410 |
| 438.0 | 0.566 | 0.157 | 0.751468 | 0.69843 | 0.716649 | 0.213005 |
| 439.5 | 0.563 | 0.154 | 0.754070 | 0.69772 | 0.716912 | 0.214685 |
| 440.5 | 0.563 | 0.152 | 0.756486 | 0.69822 | 0.718000 | 0.214073 |
| 441.5 | 0.563 | 0.150 | 0.758917 | 0.69872 | 0.719088 | 0.213469 |
| 443.5 | 0.563 | 0.147 | 0.762594 | 0.69946 | 0.720719 | 0.212578 |
| 445.5 | 0.560 | 0.147 | 0.761626 | 0.69802 | 0.719351 | 0.215209 |
| 448.0 | 0.560 | 0.145 | 0.764104 | 0.69851 | 0.720439 | 0.214625 |
| 450.5 | 0.555 | 0.145 | 0.762483 | 0.69613 | 0.718158 | 0.219050 |

Supplementary Table 2 (Continued)

| Cut-point* | Sensitivity | 1 – Specificity | Positive Predictive Value | Negative Predictive Value | Accuracy | d^2^ |
| --- | --- | --- | --- | --- | --- | --- |
| 452.5 | 0.547 | 0.143 | 0.762369 | 0.69284 | 0.715596 | 0.225658 |
| 453.5 | 0.544 | 0.143 | 0.761372 | 0.69143 | 0.714228 | 0.228385 |
| 454.5 | 0.541 | 0.143 | 0.760365 | 0.69003 | 0.712860 | 0.231130 |
| 455.5 | 0.536 | 0.143 | 0.758669 | 0.68771 | 0.710579 | 0.235745 |
| 456.5 | 0.533 | 0.143 | 0.757640 | 0.68633 | 0.709211 | 0.238538 |
| 457.5 | 0.530 | 0.143 | 0.756602 | 0.68495 | 0.707842 | 0.241349 |
| 459.0 | 0.527 | 0.141 | 0.758148 | 0.68408 | 0.707561 | 0.243610 |
| 460.5 | 0.525 | 0.136 | 0.764021 | 0.68442 | 0.709368 | 0.244121 |
| 462.0 | 0.525 | 0.134 | 0.766682 | 0.68492 | 0.710456 | 0.243581 |
| 464.5 | 0.522 | 0.134 | 0.765655 | 0.68356 | 0.709088 | 0.246440 |
| 467.0 | 0.516 | 0.134 | 0.763574 | 0.68085 | 0.706351 | 0.252212 |
| 468.5 | 0.514 | 0.131 | 0.766944 | 0.68071 | 0.707070 | 0.253357 |
| 470.0 | 0.514 | 0.129 | 0.769683 | 0.68121 | 0.708158 | 0.252837 |
| 471.5 | 0.511 | 0.129 | 0.768643 | 0.67987 | 0.706789 | 0.255762 |
| 472.5 | 0.508 | 0.129 | 0.767595 | 0.67854 | 0.705421 | 0.258705 |
| 473.5 | 0.508 | 0.127 | 0.770370 | 0.67904 | 0.706509 | 0.258193 |
| 475.0 | 0.505 | 0.127 | 0.769321 | 0.67771 | 0.705140 | 0.261154 |
| 477.0 | 0.503 | 0.127 | 0.768616 | 0.67683 | 0.704228 | 0.263138 |
| 479.5 | 0.500 | 0.127 | 0.767550 | 0.67551 | 0.702860 | 0.266129 |
| 481.5 | 0.497 | 0.127 | 0.766475 | 0.67420 | 0.701491 | 0.269138 |
| 482.5 | 0.492 | 0.127 | 0.764660 | 0.67202 | 0.699211 | 0.274193 |
| 484.5 | 0.489 | 0.127 | 0.763558 | 0.67072 | 0.697842 | 0.277250 |
| 486.5 | 0.486 | 0.127 | 0.762445 | 0.66943 | 0.696474 | 0.280325 |
| 487.5 | 0.484 | 0.127 | 0.761697 | 0.66857 | 0.695561 | 0.282385 |
| 489.5 | 0.478 | 0.127 | 0.759426 | 0.66600 | 0.692825 | 0.288613 |
| 492.0 | 0.475 | 0.127 | 0.758273 | 0.66473 | 0.691456 | 0.291754 |
| 493.5 | 0.475 | 0.124 | 0.762628 | 0.66549 | 0.693088 | 0.291001 |
| 494.5 | 0.470 | 0.124 | 0.760707 | 0.66338 | 0.690807 | 0.296276 |
| 495.5 | 0.464 | 0.124 | 0.758361 | 0.66086 | 0.688070 | 0.302672 |
| 496.5 | 0.462 | 0.124 | 0.757568 | 0.66002 | 0.687158 | 0.304820 |
| 498.0 | 0.459 | 0.124 | 0.756370 | 0.65877 | 0.685789 | 0.308057 |
| 499.5 | 0.456 | 0.124 | 0.755159 | 0.65753 | 0.684421 | 0.311312 |
| 500.5 | 0.456 | 0.122 | 0.758153 | 0.65804 | 0.685509 | 0.310820 |
| 501.5 | 0.453 | 0.122 | 0.756941 | 0.65681 | 0.684140 | 0.314093 |
| 502.5 | 0.451 | 0.122 | 0.756126 | 0.65598 | 0.683228 | 0.316285 |
| 503.5 | 0.448 | 0.122 | 0.754893 | 0.65475 | 0.681860 | 0.319588 |
| 505.0 | 0.445 | 0.118 | 0.759785 | 0.65455 | 0.682667 | 0.321949 |
| 507.5 | 0.442 | 0.111 | 0.769571 | 0.65512 | 0.685105 | 0.323685 |
| 511.0 | 0.440 | 0.111 | 0.768766 | 0.65431 | 0.684193 | 0.325921 |
| 514.0 | 0.440 | 0.108 | 0.773600 | 0.65507 | 0.685825 | 0.325264 |
| 517.5 | 0.434 | 0.108 | 0.771186 | 0.65266 | 0.683088 | 0.332020 |
| 521.0 | 0.431 | 0.108 | 0.769960 | 0.65146 | 0.681719 | 0.335425 |
| 522.5 | 0.429 | 0.108 | 0.769135 | 0.65067 | 0.680807 | 0.337705 |
| 525.0 | 0.426 | 0.108 | 0.767887 | 0.64947 | 0.679439 | 0.341140 |
| 528.0 | 0.423 | 0.108 | 0.766625 | 0.64829 | 0.678070 | 0.344593 |
| 529.5 | 0.420 | 0.108 | 0.765349 | 0.64710 | 0.676702 | 0.348064 |

Supplementary Table 2 (Continued)

| Cut-point* | Sensitivity | 1 – Specificity | Positive Predictive Value | Negative Predictive Value | Accuracy | d^2^ |
| --- | --- | --- | --- | --- | --- | --- |
| 536.5 | 0.415 | 0.108 | 0.763191 | 0.64514 | 0.674421 | 0.353889 |
| 543.0 | 0.415 | 0.106 | 0.766553 | 0.64565 | 0.675509 | 0.353461 |
| 546.0 | 0.412 | 0.106 | 0.765252 | 0.64448 | 0.674140 | 0.356980 |
| 548.5 | 0.412 | 0.104 | 0.768657 | 0.64499 | 0.675228 | 0.356560 |
| 553.5 | 0.409 | 0.104 | 0.767355 | 0.64383 | 0.673860 | 0.360097 |
| 559.0 | 0.407 | 0.104 | 0.766478 | 0.64305 | 0.672947 | 0.362465 |
| 561.5 | 0.407 | 0.101 | 0.771677 | 0.64382 | 0.674579 | 0.361850 |
| 562.5 | 0.401 | 0.099 | 0.772582 | 0.64202 | 0.672930 | 0.368602 |
| 566.0 | 0.398 | 0.099 | 0.771260 | 0.64087 | 0.671561 | 0.372205 |
| 570.5 | 0.398 | 0.097 | 0.774841 | 0.64138 | 0.672649 | 0.371813 |
| 572.5 | 0.396 | 0.097 | 0.773961 | 0.64062 | 0.671737 | 0.374225 |
| 574.0 | 0.393 | 0.097 | 0.772628 | 0.63947 | 0.670368 | 0.377858 |
| 577.5 | 0.390 | 0.094 | 0.776773 | 0.63910 | 0.670632 | 0.380936 |
| 581.0 | 0.390 | 0.090 | 0.784223 | 0.64012 | 0.672807 | 0.380200 |
| 584.5 | 0.390 | 0.088 | 0.788001 | 0.64062 | 0.673895 | 0.379844 |
| 588.0 | 0.390 | 0.085 | 0.793738 | 0.64138 | 0.675526 | 0.379325 |
| 589.5 | 0.387 | 0.085 | 0.792471 | 0.64025 | 0.674158 | 0.382994 |
| 590.5 | 0.387 | 0.083 | 0.796359 | 0.64075 | 0.675246 | 0.382658 |
| 592.0 | 0.385 | 0.083 | 0.795518 | 0.64000 | 0.674333 | 0.385114 |
| 595.0 | 0.382 | 0.083 | 0.794242 | 0.63888 | 0.672965 | 0.388813 |
| 599.5 | 0.382 | 0.081 | 0.798200 | 0.63938 | 0.674053 | 0.388485 |
| 603.0 | 0.382 | 0.078 | 0.804211 | 0.64013 | 0.675684 | 0.388008 |
| 605.0 | 0.376 | 0.078 | 0.801706 | 0.63791 | 0.672947 | 0.395460 |
| 606.5 | 0.374 | 0.078 | 0.800857 | 0.63717 | 0.672035 | 0.397960 |
| 607.5 | 0.368 | 0.078 | 0.798265 | 0.63496 | 0.669298 | 0.405508 |
| 609.0 | 0.365 | 0.078 | 0.796943 | 0.63386 | 0.667930 | 0.409309 |
| 613.0 | 0.363 | 0.078 | 0.796053 | 0.63313 | 0.667018 | 0.411853 |
| 616.5 | 0.363 | 0.076 | 0.800237 | 0.63363 | 0.668105 | 0.411545 |
| 617.5 | 0.363 | 0.074 | 0.804466 | 0.63413 | 0.669193 | 0.411245 |
| 621.5 | 0.363 | 0.071 | 0.810894 | 0.63488 | 0.670825 | 0.410810 |
| 625.5 | 0.363 | 0.069 | 0.815237 | 0.63538 | 0.671912 | 0.410530 |
| 626.5 | 0.360 | 0.069 | 0.813984 | 0.63429 | 0.670544 | 0.414361 |
| 627.5 | 0.360 | 0.067 | 0.818396 | 0.63479 | 0.671632 | 0.414089 |
| 630.0 | 0.354 | 0.067 | 0.815885 | 0.63263 | 0.668895 | 0.421805 |
| 632.5 | 0.352 | 0.067 | 0.815033 | 0.63191 | 0.667982 | 0.424393 |
| 633.5 | 0.352 | 0.065 | 0.819558 | 0.63240 | 0.669070 | 0.424129 |
| 635.0 | 0.349 | 0.065 | 0.818288 | 0.63133 | 0.667702 | 0.428026 |
| 637.5 | 0.346 | 0.065 | 0.817001 | 0.63026 | 0.666333 | 0.431941 |
| 639.5 | 0.341 | 0.065 | 0.814815 | 0.62848 | 0.664053 | 0.438506 |
| 641.5 | 0.338 | 0.065 | 0.813478 | 0.62742 | 0.662684 | 0.442469 |
| 644.0 | 0.327 | 0.065 | 0.808405 | 0.62356 | 0.657667 | 0.457154 |
| 645.5 | 0.324 | 0.065 | 0.806974 | 0.62252 | 0.656298 | 0.461201 |
| 647.0 | 0.324 | 0.062 | 0.814228 | 0.62327 | 0.657930 | 0.460820 |
| 648.5 | 0.319 | 0.062 | 0.811864 | 0.62154 | 0.655649 | 0.467605 |
| 649.5 | 0.313 | 0.062 | 0.808946 | 0.61947 | 0.652912 | 0.475813 |
| 651.0 | 0.313 | 0.060 | 0.813963 | 0.61997 | 0.654000 | 0.475569 |

Supplementary Table 2 (Continued)

| Cut-point* | Sensitivity | 1 – Specificity | Positive Predictive Value | Negative Predictive Value | Accuracy | d^2^ |
| --- | --- | --- | --- | --- | --- | --- |
| 654.0 | 0.310 | 0.058 | 0.81761 | 0.61945 | 0.653719 | 0.479464 |
| 656.5 | 0.308 | 0.058 | 0.816643 | 0.61877 | 0.652807 | 0.482228 |
| 659.5 | 0.308 | 0.055 | 0.824462 | 0.61951 | 0.654439 | 0.481889 |
| 662.0 | 0.308 | 0.053 | 0.829759 | 0.62001 | 0.655526 | 0.481673 |
| 663.5 | 0.305 | 0.053 | 0.828371 | 0.61899 | 0.654158 | 0.485834 |
| 664.5 | 0.302 | 0.053 | 0.826962 | 0.61798 | 0.652789 | 0.490013 |
| 665.5 | 0.299 | 0.053 | 0.825528 | 0.61696 | 0.651421 | 0.494210 |
| 667.0 | 0.297 | 0.053 | 0.824560 | 0.61629 | 0.650509 | 0.497018 |
| 670.0 | 0.294 | 0.053 | 0.823086 | 0.61528 | 0.649140 | 0.501245 |
| 672.5 | 0.291 | 0.053 | 0.821588 | 0.61428 | 0.647772 | 0.505490 |
| 675.0 | 0.288 | 0.053 | 0.820064 | 0.61328 | 0.646404 | 0.509753 |
| 678.0 | 0.286 | 0.053 | 0.819033 | 0.61261 | 0.645491 | 0.512605 |
| 679.5 | 0.280 | 0.051 | 0.821578 | 0.61113 | 0.643842 | 0.521001 |
| 683.5 | 0.277 | 0.046 | 0.834724 | 0.61139 | 0.645193 | 0.524845 |
| 687.5 | 0.275 | 0.044 | 0.839793 | 0.61123 | 0.645368 | 0.527561 |
| 688.5 | 0.272 | 0.044 | 0.838312 | 0.61025 | 0.644000 | 0.531920 |
| 690.0 | 0.269 | 0.044 | 0.836803 | 0.60927 | 0.642632 | 0.536297 |
| 694.0 | 0.264 | 0.044 | 0.834225 | 0.60764 | 0.640351 | 0.543632 |
| 697.5 | 0.261 | 0.044 | 0.832638 | 0.60667 | 0.638982 | 0.548057 |
| 699.0 | 0.258 | 0.044 | 0.831021 | 0.60571 | 0.637614 | 0.552500 |
| 700.5 | 0.255 | 0.044 | 0.829372 | 0.60474 | 0.636246 | 0.556961 |
| 701.5 | 0.250 | 0.044 | 0.826551 | 0.60314 | 0.633965 | 0.564436 |
| 702.5 | 0.247 | 0.044 | 0.824814 | 0.60219 | 0.632596 | 0.568945 |
| 704.5 | 0.245 | 0.044 | 0.823636 | 0.60155 | 0.631684 | 0.571961 |
| 706.5 | 0.242 | 0.044 | 0.821839 | 0.60060 | 0.630316 | 0.576500 |
| 707.5 | 0.239 | 0.044 | 0.820005 | 0.59965 | 0.628947 | 0.581057 |
| 710.5 | 0.236 | 0.044 | 0.818133 | 0.59871 | 0.627579 | 0.585632 |
| 717.0 | 0.236 | 0.041 | 0.828406 | 0.59946 | 0.629211 | 0.585377 |
| 723.0 | 0.234 | 0.041 | 0.827192 | 0.59883 | 0.628298 | 0.588437 |
| 725.5 | 0.231 | 0.041 | 0.825340 | 0.59789 | 0.626930 | 0.593042 |
| 729.5 | 0.231 | 0.039 | 0.832432 | 0.59839 | 0.628018 | 0.592882 |
| 733.5 | 0.228 | 0.039 | 0.830601 | 0.59746 | 0.626649 | 0.597505 |
| 735.5 | 0.225 | 0.039 | 0.828729 | 0.59652 | 0.625281 | 0.602146 |
| 738.0 | 0.223 | 0.039 | 0.827458 | 0.59590 | 0.624368 | 0.605250 |
| 740.5 | 0.220 | 0.039 | 0.825516 | 0.59498 | 0.623000 | 0.609921 |
| 747.0 | 0.217 | 0.039 | 0.823529 | 0.59405 | 0.621632 | 0.614610 |
| 754.0 | 0.214 | 0.039 | 0.821497 | 0.59313 | 0.620263 | 0.619317 |
| 757.0 | 0.212 | 0.039 | 0.820116 | 0.59251 | 0.619351 | 0.622465 |
| 761.5 | 0.206 | 0.039 | 0.815842 | 0.59068 | 0.616614 | 0.631957 |
| 765.5 | 0.206 | 0.037 | 0.823620 | 0.59118 | 0.617702 | 0.631805 |
| 766.5 | 0.203 | 0.037 | 0.821479 | 0.59027 | 0.616333 | 0.636578 |
| 768.0 | 0.201 | 0.037 | 0.820022 | 0.58967 | 0.615421 | 0.639770 |
| 769.5 | 0.201 | 0.035 | 0.828078 | 0.59017 | 0.616509 | 0.639626 |
| 773.5 | 0.198 | 0.035 | 0.825927 | 0.58926 | 0.615140 | 0.644429 |
| 778.0 | 0.195 | 0.035 | 0.823721 | 0.58836 | 0.613772 | 0.649250 |
| 781.0 | 0.192 | 0.035 | 0.821458 | 0.58746 | 0.612404 | 0.654089 |

Supplementary Table 2 (Continued)

| Cut-point* | Sensitivity | 1 – Specificity | Positive Predictive Value | Negative Predictive Value | Accuracy | d^2^ |
| --- | --- | --- | --- | --- | --- | --- |
| 786.0 | 0.190 | 0.032 | 0.832771 | 0.58761 | 0.613123 | 0.657124 |
| 788.0 | 0.187 | 0.032 | 0.830543 | 0.58671 | 0.611754 | 0.661993 |
| 789.5 | 0.184 | 0.032 | 0.828255 | 0.58582 | 0.610386 | 0.666880 |
| 792.5 | 0.181 | 0.032 | 0.825904 | 0.58493 | 0.609018 | 0.671785 |
| 796.5 | 0.181 | 0.030 | 0.834989 | 0.58543 | 0.610105 | 0.671661 |
| 799.0 | 0.179 | 0.030 | 0.833453 | 0.58484 | 0.609193 | 0.674941 |
| 801.0 | 0.173 | 0.030 | 0.828666 | 0.58307 | 0.606456 | 0.684829 |
| 802.5 | 0.173 | 0.028 | 0.838241 | 0.58357 | 0.607544 | 0.684713 |
| 805.0 | 0.168 | 0.028 | 0.834225 | 0.58210 | 0.605263 | 0.693008 |
| 808.5 | 0.165 | 0.028 | 0.831718 | 0.58123 | 0.603895 | 0.698009 |
| 810.5 | 0.165 | 0.025 | 0.846989 | 0.58198 | 0.605526 | 0.697850 |
| 812.0 | 0.162 | 0.025 | 0.844596 | 0.58110 | 0.604158 | 0.702869 |
| 813.5 | 0.159 | 0.025 | 0.842127 | 0.58023 | 0.602789 | 0.707906 |
| 816.0 | 0.157 | 0.025 | 0.840436 | 0.57966 | 0.601877 | 0.711274 |
| 819.5 | 0.154 | 0.025 | 0.837832 | 0.57879 | 0.600509 | 0.716341 |
| 821.5 | 0.151 | 0.025 | 0.835141 | 0.57793 | 0.599140 | 0.721426 |
| 824.0 | 0.148 | 0.025 | 0.832360 | 0.57707 | 0.597772 | 0.726529 |
| 829.0 | 0.146 | 0.025 | 0.830453 | 0.57649 | 0.596860 | 0.729941 |
| 833.0 | 0.146 | 0.023 | 0.841872 | 0.57699 | 0.597947 | 0.729845 |
| 835.0 | 0.143 | 0.023 | 0.839088 | 0.57614 | 0.596579 | 0.734978 |
| 836.5 | 0.140 | 0.023 | 0.836205 | 0.57528 | 0.595211 | 0.740129 |
| 838.0 | 0.140 | 0.021 | 0.848287 | 0.57578 | 0.596298 | 0.740041 |
| 840.0 | 0.137 | 0.021 | 0.845478 | 0.57493 | 0.594930 | 0.745210 |
| 842.0 | 0.135 | 0.021 | 0.843547 | 0.57437 | 0.594018 | 0.748666 |
| 847.0 | 0.132 | 0.021 | 0.840558 | 0.57352 | 0.592649 | 0.753865 |
| 852.0 | 0.126 | 0.021 | 0.834225 | 0.57184 | 0.589912 | 0.764317 |
| 857.0 | 0.124 | 0.021 | 0.832000 | 0.57128 | 0.589000 | 0.767817 |
| 861.5 | 0.121 | 0.021 | 0.828549 | 0.57044 | 0.587632 | 0.773082 |
| 863.0 | 0.118 | 0.021 | 0.824953 | 0.56960 | 0.586263 | 0.778365 |
| 866.0 | 0.113 | 0.021 | 0.818612 | 0.56822 | 0.583982 | 0.787210 |
| 872.5 | 0.110 | 0.021 | 0.814583 | 0.56739 | 0.582614 | 0.792541 |
| 878.0 | 0.107 | 0.021 | 0.810370 | 0.56656 | 0.581246 | 0.797890 |
| 884.0 | 0.104 | 0.021 | 0.805961 | 0.56574 | 0.579877 | 0.803257 |
| 892.5 | 0.104 | 0.018 | 0.828939 | 0.56649 | 0.581509 | 0.803140 |
| 896.5 | 0.102 | 0.018 | 0.826168 | 0.56594 | 0.580596 | 0.806728 |
| 897.5 | 0.096 | 0.018 | 0.817289 | 0.56431 | 0.577860 | 0.817540 |
| 902.5 | 0.093 | 0.018 | 0.812500 | 0.56349 | 0.576491 | 0.822973 |
| 908.0 | 0.093 | 0.016 | 0.829787 | 0.56399 | 0.577579 | 0.822905 |
| 912.0 | 0.091 | 0.016 | 0.826695 | 0.56345 | 0.576667 | 0.826537 |
| 915.5 | 0.088 | 0.016 | 0.821839 | 0.56264 | 0.575298 | 0.832000 |
| 916.5 | 0.085 | 0.016 | 0.816704 | 0.56183 | 0.573930 | 0.837481 |
| 921.0 | 0.082 | 0.014 | 0.830865 | 0.56152 | 0.573649 | 0.842920 |
| 929.0 | 0.080 | 0.014 | 0.827367 | 0.56099 | 0.572737 | 0.846596 |
| 934.5 | 0.077 | 0.014 | 0.821839 | 0.56019 | 0.571368 | 0.852125 |
| 937.0 | 0.074 | 0.014 | 0.815946 | 0.55939 | 0.570000 | 0.857672 |
| 938.5 | 0.071 | 0.014 | 0.809649 | 0.55859 | 0.568632 | 0.863237 |

Supplementary Table 2 (Continued)

| Cut-point* | Sensitivity | 1 – Specificity | Positive Predictive Value | Negative Predictive Value | Accuracy | d^2^ |
| --- | --- | --- | --- | --- | --- | --- |
| 961.5 | 0.063 | 0.014 | 0.790541 | 0.55647 | 0.564982 | 0.878165 |
| 965.0 | 0.060 | 0.014 | 0.782347 | 0.55568 | 0.563614 | 0.883796 |
| 977.5 | 0.058 | 0.014 | 0.776519 | 0.55516 | 0.562702 | 0.887560 |
| 988.5 | 0.055 | 0.014 | 0.767167 | 0.55437 | 0.561333 | 0.893221 |
| 995.5 | 0.052 | 0.014 | 0.756999 | 0.55359 | 0.559965 | 0.898900 |
| 1003.0 | 0.049 | 0.014 | 0.745902 | 0.55281 | 0.558596 | 0.904597 |
| 1008.0 | 0.047 | 0.014 | 0.737923 | 0.55229 | 0.557684 | 0.908405 |
| 1017.0 | 0.044 | 0.014 | 0.724968 | 0.55151 | 0.556316 | 0.914132 |
| 1028.5 | 0.041 | 0.014 | 0.710667 | 0.55074 | 0.554947 | 0.919877 |
| 1037.0 | 0.038 | 0.014 | 0.694796 | 0.54997 | 0.553579 | 0.925640 |
| 1047.0 | 0.036 | 0.014 | 0.683212 | 0.54945 | 0.552667 | 0.929492 |
| 1056.5 | 0.033 | 0.014 | 0.664087 | 0.54868 | 0.551298 | 0.935285 |
| 1077.0 | 0.030 | 0.014 | 0.642504 | 0.54792 | 0.549930 | 0.941096 |
| 1097.5 | 0.027 | 0.014 | 0.617958 | 0.54715 | 0.548561 | 0.946925 |
| 1113.0 | 0.025 | 0.014 | 0.599631 | 0.54664 | 0.547649 | 0.950821 |
| 1132.5 | 0.025 | 0.012 | 0.636008 | 0.54714 | 0.548737 | 0.950769 |
| 1166.5 | 0.022 | 0.012 | 0.605932 | 0.54638 | 0.547368 | 0.956628 |
| 1196.5 | 0.019 | 0.012 | 0.570439 | 0.54562 | 0.546000 | 0.962505 |
| 1210.0 | 0.016 | 0.012 | 0.527919 | 0.54487 | 0.544632 | 0.968400 |
| 1245.5 | 0.014 | 0.012 | 0.494565 | 0.54436 | 0.543719 | 0.972340 |
| 1273.5 | 0.014 | 0.009 | 0.566096 | 0.54511 | 0.545351 | 0.972277 |
| 1371.5 | 0.014 | 0.007 | 0.626506 | 0.54561 | 0.546439 | 0.972245 |
| 1500.5 | 0.014 | 0.005 | 0.701349 | 0.54611 | 0.547526 | 0.972221 |
| 1587.0 | 0.011 | 0.005 | 0.648526 | 0.54536 | 0.546158 | 0.978146 |
| 1740.0 | 0.011 | 0.002 | 0.821839 | 0.54611 | 0.547789 | 0.978125 |
| 2028.0 | 0.011 | 0.000 | 1.000000 | 0.54660 | 0.548877 | 0.978121 |
| 2247.5 | 0.008 | 0.000 | 1.000000 | 0.54585 | 0.547509 | 0.984064 |
| 2342.5 | 0.005 | 0.000 | 1.000000 | 0.54510 | 0.546140 | 0.990025 |
| 2530.0 | 0.003 | 0.000 | 1.000000 | 0.54460 | 0.545228 | 0.994009 |
| 2657.0 | 0.000 | 0.000 | 0.000000 | 0.54386 | 0.543860 | 1.000000 |
